# Markers of Kidney Function in Early Childhood and Association With Maternal Comorbidity

**DOI:** 10.1001/jamanetworkopen.2022.43146

**Published:** 2022-11-21

**Authors:** Rikke Mohr Lytsen, Sofie Taageby Nielsen, Malene Kongsgaard Hansen, Nina Strandkjær, Ida Juul Rasmussen, Anna Axelsson Raja, R. Ottilia Vøgg, Anne-Sophie Sillesen, Pia R. Kamstrup, Ida Maria Schmidt, Kasper Iversen, Henning Bundgaard, Ruth Frikke-Schmidt

**Affiliations:** 1 Department of Clinical Biochemistry, Copenhagen University Hospital–Rigshospitalet, Copenhagen, Denmark; 2 Department of Cardiology, Copenhagen University Hospital–Rigshospitalet, Copenhagen, Denmark; 3 Department of Cardiology, Copenhagen University Hospital–Herlev-Gentofte, Copenhagen, Denmark; 4 Department of Clinical Biochemistry, Copenhagen University Hospital–Herlev-Gentofte, Copenhagen, Denmark; 5 Department of Paediatrics and Adolescent Medicine, Copenhagen University Hospital–Rigshospitalet, Copenhagen, Denmark; 6 Department of Clinical Medicine, University of Copenhagen, Copenhagen, Denmark

## Abstract

**Importance:**

Kidney functional capacity is low at birth but doubles during the first 2 weeks of life and reaches near-adult levels at age 1 to 2 years. Existing reference intervals for markers of kidney function in newborns are mostly based on preterm newborns, newborns with illness, or small cohorts of term newborns, and the consequences of maternal comorbidities for newborn kidney function are sparsely described.

**Objective:**

To establish robust reference intervals for creatinine and urea in healthy children in early childhood and to assess whether maternal comorbidity is associated with newborn creatinine and urea concentrations.

**Design, Setting, and Participants:**

This multicenter, prospective, population-based cohort study assessed data and umbilical cord blood samples from participants in the Copenhagen Baby Heart Study (CBHS) who were born between April 1, 2016, and October 31, 2018, and venous blood samples from a subsample of CBHS participants who were enrolled in the COMPARE study between May 3, 2017, and November 4, 2018. Cord blood samples of 13 354 newborns from the CBHS and corresponding venous blood samples of 444 of those newborns from the COMPARE study were included. Blood samples were collected at birth, age 2 months, and age 14 to 16 months, with follow-up completed on February 12, 2020. Healthy nonadmitted term newborns from maternity wards at 3 hospitals in the Capital Region of Denmark were included.

**Exposures:**

Maternal comorbidity.

**Main Outcomes and Measures:**

Creatinine and urea concentrations.

**Results:**

Among 13 354 newborns in the CBHS cohort, characteristics of 12 938 children were stratified by sex and gestational age (GA). Of those, 6567 children (50.8%) were male; 5259 children (40.6%) were born at 37 to 39 weeks’ GA, and 7679 children (59.4%) were born at 40 to 42 weeks’ GA. Compared with children born at 40 to 42 weeks’ GA, those born at 37 to 39 weeks’ GA had lower birth weight, Apgar scores at 5 minutes, placental weight, and placental-fetal weight ratio. Children born at 37 to 39 weeks’ GA vs those born at 40 to 42 weeks’ GA were more frequently small for GA at birth and more likely to have placental insufficiency and exposure to maternal preeclampsia, maternal diabetes, maternal kidney disease, and maternal hypertension. Among children born at 37 to 39 weeks’ GA, reference intervals were 0.54 to 1.08 mg/dL for creatinine and 5.32 to 14.67 mg/dL for urea; among children born at 40 to 42 weeks’ GA, reference intervals were 0.57 to 1.19 mg/dL for creatinine and 5.60 to 14.85 mg/dL for urea. At birth, multifactorially adjusted odds ratios among children exposed to preeclampsia were 9.40 (95% CI, 1.68-52.54) for a venous creatinine concentration higher than the upper reference limit, 4.29 (95% CI, 1.32-13.93) for a venous creatinine concentration higher than the 90th percentile, and 3.10 (95% CI, 1.14-8.46) for a venous creatinine concentration higher than the 80th percentile.

**Conclusions and Relevance:**

In this study, improved reference intervals for creatinine and urea concentrations were generated. Preeclampsia was associated with an increased risk of high newborn creatinine concentrations, suggesting that newborns of mothers with preeclampsia need closer observation of their kidney function.

## Key Points

**Question:**

What are the reference intervals for creatinine and urea concentrations in healthy newborns, and is maternal comorbidity associated with the concentrations?

**Findings:**

In this cohort study including 13 354 newborns with umbilical cord blood samples and 444 children with newborn venous blood samples, more robust and gestational age–dependent reference intervals were generated for creatinine and urea in the first 16 months of life. Furthermore, maternal preeclampsia was associated with a significant 9-fold increase in the risk of high newborn creatinine concentrations.

**Meaning:**

These novel findings have the potential to improve clinical decision-making and increase understanding of the consequences of maternal comorbidities for kidney function among newborns and infants.

## Teaser

This cohort study uses umbilical and venous blood samples from children in the Copenhagen Baby Heart Study and COMPARE study to create new reference intervals for creatinine and urea and assess the association of maternal comorbidity with newborn creatinine and urea concentrations.

## Introduction

Kidney functional capacity is low at birth but doubles during the first 2 weeks of life and reaches near-adult values at age 1 to 2 years.^1,2,3^ Existing reference intervals for markers of kidney function, such as creatinine and urea, in newborns are mostly based on findings in preterm newborns or newborns with illness (ranging from 38-580 newborns) or in small cohorts of term newborns (ranging from 5-74 newborns)^4,5,6,7,8,9,10,11,12,13,14,15,16,17,18,19,20^; hence, use of existing reference intervals may present challenges for clinical decision-making. In addition, the burden of maternal comorbidities, such as preeclampsia, diabetes, hypertension, and overweight, has increased during the last several decades, and knowledge of the implications of these factors for neonatal kidney function is sparse.^21,22^

Assessment of kidney function is important for the detection of kidney disease and acute kidney injury (AKI) and for the ability to offer safe pharmacological treatment to neonates given that approximately 60% of all drugs are eliminated by the kidneys.^23^ Kidney functional capacity is low during fetal life, and homeostasis is sustained by the placenta.^1,24,25^ During the first weeks of life, kidney function increases rapidly and reaches 90% of adult levels at age 1 year.^3^ Serum creatinine is used as a crude measure of kidney function, refined by different formulas for estimated glomerular filtration rates (including creatinine, blood urea nitrogen, cystatin C, or height).^14^ During fetal life, creatinine and urea are transferred across the placental barrier, and fetal and newborn concentrations have been reported to correlate significantly with maternal concentrations.^9,26,27,28,29,30^ During the first few days of life, newborn creatinine concentration increases,^7,12,17^ leaving the reliability of creatinine as a marker of kidney function disputed in this early phase of life. An optimal marker is, however, still lacking.^2,9,12,17^ Cystatin C and β-trace protein have been suggested as alternative markers, but testing is costly and of limited availability, and the superiority of cystatin C has been questioned.^2,9^ The development of creatinine and urea concentrations over time during early life is not well described in healthy unselected nonhospitalized children. Furthermore, the consequences of maternal comorbidities during pregnancy for newborn kidney function are currently unknown.

Therefore, we evaluated creatinine and urea concentrations in the first 16 months of life in umbilical cord blood and venous blood samples from healthy, nonadmitted newborns to define robust reference intervals at birth stratified by gestational age (GA). Furthermore, we examined the association of maternal comorbidities with creatinine and urea concentrations in newborns. The study included 13 354 newborns with umbilical cord blood samples from the prospective population-based Copenhagen Baby Heart Study (CBHS). Among those, 444 newborns included in the COMPARE study had corresponding venous blood samples collected at birth, 363 infants had venous samples collected at age 2 months, and 156 children had venous samples collected at age 14 to 16 months.

## Methods

The CBHS and the COMPARE study adhered to the Declaration of Helsinki^31^ and were approved by the Regional Ethics Committee of the Capital Region of Denmark and the Danish Data Protection Agency. For all participants, written informed consent from legal guardians was obtained. Both studies followed the Strengthening the Reporting of Observational Studies in Epidemiology (STROBE) reporting guideline for cohort studies.

### Copenhagen Baby Heart Study

The CBHS was a prospective multicenter population-based cohort study focusing on cardiac structure and function in 27 595 newborns and has been previously described in detail^32,33^; follow-up of some participants is ongoing. Children born between April 1, 2016, and October 31, 2018, at Copenhagen University Hospital–Rigshospitalet (Rigshospitalet), Herlev Hospital, or Hvidovre Hospital were prenatally included in the study.^33^ Umbilical cord blood samples were collected immediately after birth in accordance with the CBHS study protocol.^32^ The CBHS had a high participation rate, with 55% of infants born in participating hospitals enrolled.^33^ Data on participant race and ethnicity were not included in the current study because analyses were primarily stratified by sex and GA; however, Vøgg et al^33^ reported that 78.9% of children in the CBHS had at least 1 parent with Danish citizenship, and 21.2% of mothers were of origins other than Denmark.

### COMPARE Study

Mothers enrolled in the CBHS who delivered at Rigshospitalet were also offered enrollment in the COMPARE study between May 3, 2017, and November 4, 2018. Exclusion criteria for the COMPARE study were birth at less than 37 weeks’ GA or birth at 42 weeks’ GA or greater and birth weight lower than 2500 g. In this subcohort of the CBHS, the sampling of umbilical cord blood was followed by parallel sampling of venous blood from the newborn, mother, and father from the antecubital vein approximately 2 hours after birth. The collection of venous blood samples was repeated at 2 months after birth in infants and their parents and again at age 14 to 16 months in children and their mothers.^34^ Follow-up was completed on February 12, 2020.

### Biochemical Measurements and Covariates

Umbilical blood and venous blood samples were analyzed for biochemical markers, including creatinine and urea, using standard hospital clinical biochemistry equipment.^32^ Creatinine and urea concentrations are reported in milligrams per deciliter (to convert creatinine from milligrams per deciliter to micromoles per liter, multiply by 88.4; to convert urea from milligrams per deciliter to millimoles per liter, multiply by 0.357). Additional information about biochemical measurements is provided in the eMethods in the Supplement.

Demographic and clinical characteristics of mothers and newborns were extracted from the local obstetric and fetal medicine databases. Maternal age was recorded at delivery. Maternal body mass index (BMI; calculated as weight in kilograms divided by height in meters squared) was self-reported and calculated using weight before pregnancy. Gestational age was determined from obstetric ultrasonography. Neonatal Apgar (appearance, pulse, grimace, activity, and respiration) scores were assessed at 1 minute and 5 minutes after birth using a scale of 1 to 10, with scores of 7 to 10 considered good to excellent health. Birth weight and length were measured at birth; neonates who were small for GA (SGA) at birth were identified using the formula of Marsál et al,^35^ with SGA defined as birth weight lower than the 2.3 percentile and equivalent to a birth weight less than 22% of the mean birth weight for a given GA. The placental-fetal weight ratio was calculated as placental weight divided by birth weight and reported as grams of placental weight per 1 gram of fetal weight.

Maternal and newborn diagnoses were identified using *International Statistical Classification of Diseases and Related Health Problems, Tenth Revision *(*ICD-10*) diagnostic codes. Relevant comorbidities included placental insufficiency, congenital malformation of the urinary system, and maternal preeclampsia, diabetes, hypertension, and kidney disease. Information on smoking was self-reported during a prepregnancy interview with a midwife during the first trimester. (*ICD-10* diagnosis codes are provided in eTable 1 in the Supplement.)

### Statistical Analysis

Statistical analyses were conducted using Stata software, version 17.0 (StataCorp LLC), and R Studio software, version 3.6 (R Foundation for Statistical Computing). Categorical variables are reported as numbers and percentages, and continuous variables are reported as medians and IQRs. Two-sided *P* < .05 was considered statistically significant. The Mann-Whitney *U* test was applied for 2-group comparisons of unmatched continuous variables, and the Pearson χ^2^ test was applied for unmatched categorical variables. Unpaired *t* tests were used to compare maternal venous blood with umbilical cord blood and newborn venous blood, and paired *t* tests were used to compare umbilical cord blood with newborn venous blood. Pearson correlation coefficients and linear regression analyses were performed to describe the association between newborn venous blood and umbilical cord blood.

Reference intervals for concentrations in children’s venous blood at birth, 2 months after birth, and 14 to 16 months after birth were established using the nonparametric method recommended in the *Tietz Textbook of Clinical Chemistry and Molecular Diagnostics*^36^ for laboratories of clinical biochemistry. Thus, outliers (defined as values outside of the first quartile minus 1.5 multiplied by IQR and values outside of the third quartile plus 1.5 multiplied by IQR) were removed. The 2.5 and 97.5 percentiles were defined using 4 different models: (1) no exclusions, (2) outliers removed according to the Tietz method,^36^ (3) individuals with exclusion criteria removed, and (4) both outliers and individuals with exclusion criteria removed. The exclusion criteria used in models 3 and 4 were defined as any record of preeclampsia, maternal diabetes, maternal kidney disease, congenital kidney deformity (diagnosis codes are available in eTable 1 in the Supplement), maternal BMI greater than 35, and SGA at birth.^35^ Newborn reference intervals were stratified by GA.

The association of maternal comorbidities with creatinine and urea concentrations in children were examined using logistic regression analysis, with maternal comorbidities as exposures and high creatinine and urea at different cutoffs as end points. The models were adjusted for maternal age at birth, child’s sex, GA, birth weight, birth length, multiple births, and placental-fetal weight ratio higher or lower than the median. Sensitivity analyses additionally adjusted for maternal creatinine and urea concentrations in the COMPARE study were also performed.

## Results

### Study Participants

A flowchart of the enrollment process, number of participants, and blood samples collected in the CBHS and COMPARE studies is shown in eFigure 1 in the Supplement. The current study included umbilical cord blood samples from 13 354 newborns, with 444 parallel newborn venous blood samples collected at birth, 363 venous samples collected at age 2 months, and 156 venous samples collected at age 14 to 16 months.

### Characteristics of the Study Population

Characteristics of 12 938 children in the CBHS and 444 children in the COMPARE study, stratified by sex and GA, are shown in Table 1. Among 12 938 children in the CBHS cohort, 6371 children (49.2%) were female, 6567 children (50.8%) were male; 5259 children (40.6%) were born at 37 to 39 weeks’ GA, and 7679 children (59.4%) were born at 40 to 42 weeks’ GA. The median maternal age in the CBHS cohort was 31 years (IQR, 29-35 years), and the median maternal BMI was 22 (IQR, 21-25). Additional characteristics, such as maternal socioeconomic status, educational level, and ethnicity, have previously been described in detail.^33^ Most children (78.9%) in the CBHS had at least 1 parent with Danish citizenship.^33^ Values for some variables were missing among 1711 of 13 354 participants in the CBHS cohort. An assessment of missing values is shown in eTable 2 in the Supplement.

**Table 1.** Characteristics of Participants in the Copenhagen Baby Heart Study and the COMPARE Study

Characteristic^a^	Female participants, No./total No. (%)			Male participants, No./total No. (%)		
	GA, wk^b^		*P* value^c^	GA, wk^b^		*P* value^c^
	37-39	40-42		37-39	40-42	
**Copenhagen Baby Heart Study** ^d^						
Participants	2521/12 938 (19.5)	3850/12 938 (29.8)	NA	2738/12 938 (21.2)	3829/12 938 (29.6)	NA
Maternal age, median (IQR), y^e^	32 (29-35)	31 (29-34)	<.001	32 (29-35)	31 (29-35)	.008
Birth weight, median (IQR), g^f^	3299 (3028-3580)	3601 (3324-3896)	<.001	3420 (3123-3699)	3758 (3480-4056)	<.001
Birth length, median (IQR), cm^f^	51 (49-52)	52 (51-53)	<.001	51 (50-52)	53 (52-54)	<.001
Apgar score at 5 min, median (IQR)^g^	10 (10-10)	10 (10-10)	.004	10 (10-10)	10 (10-10)	.31
SGA^h^	89/2521 (3.5)	98/3850 (2.5)	.02	91/2738 (3.3)	92/3829 (2.4)	.03
Placental weight, median (IQR), g	615 (530-710)	640 (553-724)	<.001	630 (540-725)	650 (575-750)	<.001
Placental-fetal weight ratio, median (IQR), g/1 g of fetal weight^i^	0.19 (0.17-0.21)	0.18 (0.16-0.20)	<.001	0.18 (0.16-0.21)	0.17 (0.16-0.19)	<.001
Placental insufficiency^j^	203/2518 (8.1)	142/3850 (3.7)	<.001	194/2735 (7.1)	82/3829 (2.1)	<.001
Congenital malformation of the urinary system^j^	3/2512 (0.1)	3/3848 (0.1)	.60	5/2726 (0.2)	11/3818 (0.3)	.40
Maternal BMI, median (IQR)^k^	23 (21-25)	22 (21-25)	.18	22 (21-25)	22 (21-25)	.21
Maternal comorbidities^j^						
Preeclampsia	190/2518 (7.5)	147/3850 (3.8)	<.001	222/2735 (8.1)	112/3829 (2.9)	<.001
Diabetes	119/2518 (4.7)	36/3850 (0.9)	<.001	144/2735 (5.3)	48/3829 (1.3)	<.001
Kidney disease	5/2518 (0.2)	0	.006	4/2735 (0.1)	2/3829 (0.1)	.21
Hypertension	100/2518 (4.0)	71/3850 (1.8)	<.001	118/2735 (4.3)	53/3829 (1.4)	<.001
Smoking^l^	86/2478 (3.5)	107/3763 (2.9)	.14	88/2691 (3.3)	108/3747 (2.9)	.42
**COMPARE study** ^d^						
Participants	106/444 (23.9)	111/444 (25.0)	NA	105/444 (23.6)	122/444 (27.5)	NA
Maternal age, median (IQR), y^e^	33 (30-37)	32 (29-35)	.10	33 (30-37)	31 (28-34)	.006
Birth weight, median (IQR), g^f^	3230 (3020-3521)	3521 (3335-3779)	<.001	3418 (3166-3559)	3746 (3494-4107)	<.001
Birth length, median (IQR), cm^f^	51 (50-52)	52 (51-53)	<.001	51 (50-52)	53 (52-55)	<.001
Apgar score at 5 min, median (IQR)^g^	10 (10-10)	10 (10-10)	.91	10 (10-10)	10 (10-10)	.89
SGA^h^	1/106 (0.9)	6/111 (5.4)	.06	4/105 (3.8)	1/122 (0.8)	.13
Placental weight, median (IQR), g	628 (565-730)	618 (540-700)	.14	660 (580-780)	660 (560-770)	.41
Placental-fetal weight ratio, median (IQR), g/1 g of fetal weight^i^	0.19 (0.18-0.22)	0.17 (0.15-0.19)	<.001	0.20 (0.17-0.21)	0.17 (0.15-0.20)	<.001
Placental insufficiency^j^	7/106 (6.6)	4/111 (3.6)	.31	7/105 (6.7)	1/122 (0.8)	.02
Congenital malformation of the urinary system^j^	0	0		0	0	
Maternal BMI, median (IQR)^k^	23 (21-25)	22 (21-25)	.38	23 (20-24)	22 (21-26)	.90
Maternal comorbidities^j^						
Preeclampsia	7/106 (6.6)	5/111 (4.5)	.50	9/105 (8.6)	6/122 (4.9)	.27
Diabetes	3/106 (2.8)	0	.07	10/105 (9.5)	1/122 (0.8)	.002
Kidney disease	0	0	NA	0	0	NA
Hypertension	5/106 (4.7)	3/111 (2.7)	.43	6/105 (5.7)	6/122 (4.9)	.79
Smoking^l^	2/103 (2.0)	0	.35	0	1/119 (0.8)	.11

Abbreviations: BMI, body mass index (calculated as weight in kilograms divided by height in meters squared); GA, gestational age; NA, not applicable; SGA, small for gestational age.

^a^ Baseline characteristics on the day of birth (2016-2018) stratified by sex and GA.

^b^ Gestational age was determined from obstetric ultrasonography.

^c^ *P* values were derived from a Mann-Whitney *U* test or a Pearson χ^2^ test.

^d^ Numbers (including total number of participants in the Copenhagen Baby Heart Study) may vary from the flowchart (eFigure 1 in the Supplement) due to missing variables.

^e^ Maternal age was recorded at delivery.

^f^ Birth weight and birth length were measured at birth.

^g^ Apgar scores were assessed at 1 minute and 5 minutes after birth using a scale ranging from 1 to 10, with scores of 7 to 10 considered good to excellent health.

^h^ Small for gestational age: birth weight below the 2.3 percentile and equivalent to a birth weight less than 22% of the mean birth weight for gestational age.^35^

^i^ Placental-fetal weight ratio was calculated as placental weight divided by birth weight and reported as grams of placental weight per 1 gram of fetal weight.

^j^ Additional details about placental insufficiency, congenital malformation of the urinary system, and maternal comorbidities (preeclampsia, diabetes, kidney disease, and hypertension) are available in eTable 1 in the Supplement.

^k^ Maternal BMI was self-reported and calculated using weight before pregnancy.

^l^ Information about smoking was self-reported during a prepregnancy interview with a midwife in the first trimester.

Among 444 children in the COMPARE subcohort, 217 (48.9%) were female, and 227 (51.1%) were male; 211 children (47.5%) were born at 37 to 39 weeks’ GA, and 233 (52.5%) were born at 40 to 42 weeks’ GA. The median maternal age in the COMPARE subcohort was 32 years (IQR, 29-36 years), and the median maternal BMI was 23 (IQR, 21-25). In both cohorts, the incidence of maternal comorbidities was low (eg, preeclampsia: 671 women [5.2%] in the CBHS cohort vs 27 women [6.1%] in the COMPARE subcohort; diabetes: 347 women [2.7%] in the CBHS cohort vs 14 women [3.2%] in the COMPARE subcohort).

In the CBHS cohort, compared with children born at 40 to 42 weeks’ GA, those born at 37 to 39 weeks’ GA had lower birth weight (median [IQR], 3360 [3075-3644] g vs 3680 [3400-3980] g; *P* < .001), birth length (median [IQR], 51 [50-52] cm vs 52 [51-54] cm; *P* < .001), Apgar scores at 5 minutes (median [IQR], 10 [10-10] vs 10 [10-10]; *P* = .01), placental weight (median [IQR], 623 [534-720] g vs 648 [560-735] g; *P* < .001), and placental-fetal weight ratio (median [IQR], 0.19 [0.16-0.21] g/1 g of fetal weight vs 0.18 [0.16-0.20] g/1 g of fetal weight; *P* < .001). Children born at 37 to 39 weeks’ GA (n = 5259) vs those born at 40 to 42 weeks’ GA (n = 7679) were more frequently SGA at birth (180 children [3.4%] vs 190 children [2.5%]; *P* = .001) and more likely to have placental insufficiency (397 children [7.5%] vs 224 children [2.9%]; *P* < .001) and exposure to maternal preeclampsia (412 children [7.8%] vs 259 children [3.4%]; *P* < .001), maternal diabetes (263 children [5.0%] vs 84 children [1.1%]; *P* < .001), maternal kidney disease (9 children [0.2%] vs 2 children [0.03%]; *P* = .005), and maternal hypertension (218 children [4.1%] vs 124 children [1.6%]; *P* < .001).

### Comparison of Umbilical Cord Blood, Maternal Venous Blood, and Newborn Venous Blood at Birth

**Figure 1.**
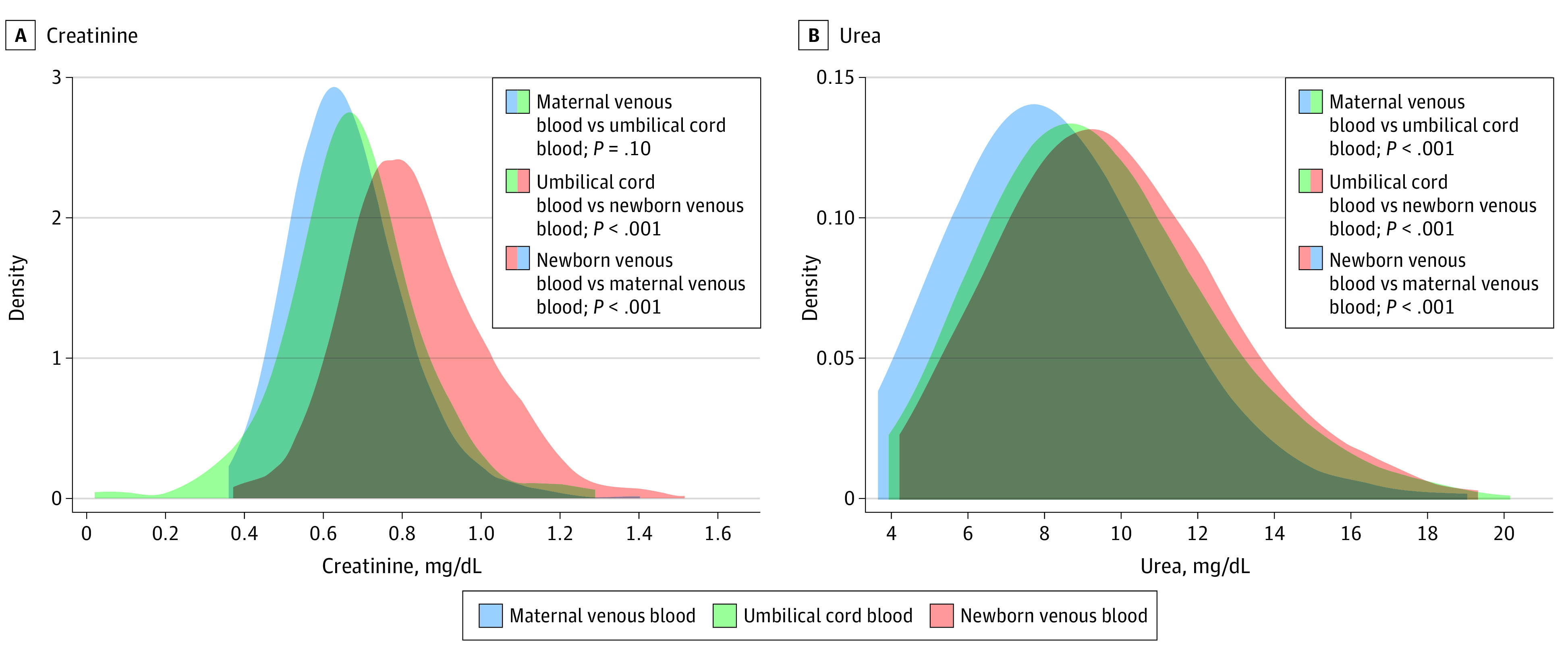
Creatinine and Urea Concentrations in Maternal Venous, Umbilical Cord, and Newborn Venous Blood at Birth The area under the density curve represents probability and therefore sums to 1. The scale of the y-axis in each density plot was determined by the range of the x-axis and ranges from 1 to infinity. *P* values were derived from unpaired *t* tests comparing maternal venous blood with newborn venous blood and umbilical cord blood. Paired *t* tests were used to compare newborn venous blood with umbilical cord blood. Equations used in the linear regression modeling of newborn venous blood as a function of umbilical cord blood, stratified by gestational age, are provided in the Results section under Comparison of Umbilical Cord Blood, Maternal Venous Blood, and Newborn Venous Blood at Birth.

The distributions of creatinine and urea concentrations in umbilical cord blood, maternal venous blood, and newborn venous blood at birth are shown in Figure 1. Median umbilical cord concentrations did not differ from maternal venous concentrations for creatinine (0.68 mg/dL vs 0.64 mg/dL; mean difference, 0.022; 95% CI, −0.002 to 0.045; *P* = .10) but were higher for urea (8.96 mg/dL vs 8.12 mg/dL; mean difference, 1.03; 95% CI, 0.63-1.42; *P* < .001). Median newborn venous concentrations at 2 hours were higher than maternal venous concentrations (creatinine: 0.80 mg/dL vs 0.64 mg/dL; mean difference, 0.17 [95% CI, 0.15-0.20; *P* < .001]; urea: 9.52 mg/dL vs 8.12 mg/dL; mean difference, 1.43 [95% CI, 1.07-1.80; *P* < .001]) and umbilical concentrations (creatinine: 0.80 mg/dL vs 0.68 mg/dL; mean difference, 0.14 [95% CI, 0.13-0.16; *P* < .001]; urea: 9.52 mg/dL vs 8.96 mg/dL; mean difference, 0.32 [95% CI, 0.24-0.40; *P* < .001]).

Pearson correlation coefficients between newborn venous blood and umbilical cord blood concentrations were 0.72 (95% CI, 0.66-0.77) for creatinine and 0.97 (95% CI, 0.96-0.97) for urea. Therefore, we created models describing the association between concentrations of creatinine and urea in newborn venous and umbilical cord blood.

For creatinine, the equations were as follows:

Newborn Cr = (0.73 × Umbilical Creatinine) + 0.33;

Newborn Cr at 37 to 39 Weeks’ GA = (0.61 × Umbilical Creatinine) + 0.40;

Newborn Cr at 40 to 42 Weeks’ GA = (0.82 × Umbilical Creatinine) + 0.27;

where Cr indicates creatinine and all values are given in mg/dL.

For urea, the equations were as follows:

Newborn Urea = (0.97 × Umbilical Urea) + 0.60;

Newborn Urea at 37 to 39 Weeks’ GA = (0.97 × Umbilical Urea) + 0.58;

Newborn Urea at 40 to 42 Weeks’ GA = (0.97 × Umbilical Urea) + 0.66,

where all values are given in mg/dL.

### Creatinine and Urea Concentrations at Birth, Age 2 Months, and Age 14 to 16 Months

**Figure 2.**
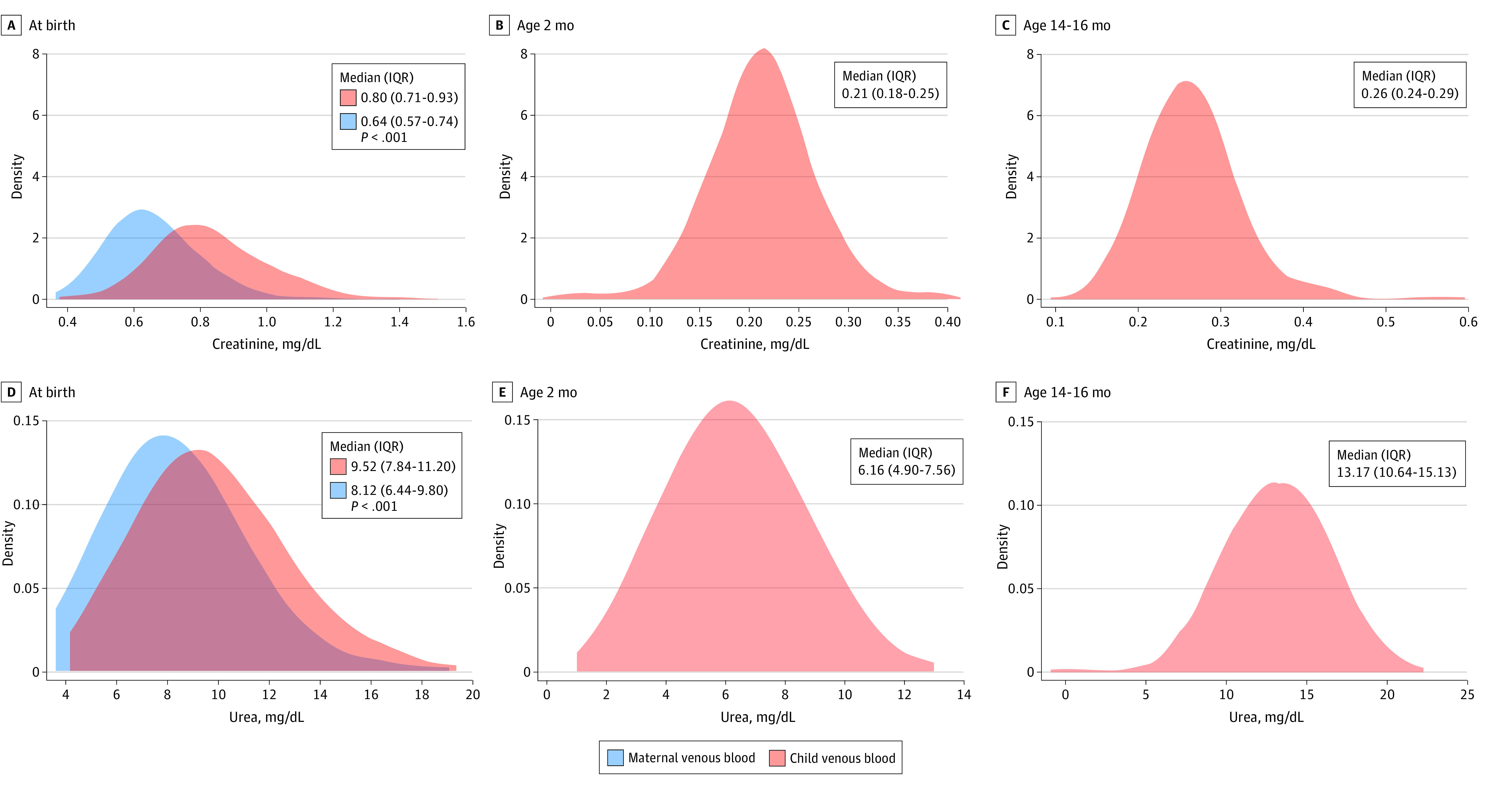
Development of Creatinine and Urea Concentrations in Venous Blood in Early Childhood The area under the density curve represents probability and therefore sums to 1. The scale of the y-axis in each density plot was determined by the range of the x-axis and ranges from 1 to infinity. *P* values were derived from unpaired *t* tests.

Changes in creatinine and urea concentrations from birth to age 2 months and birth to age 14 to 16 months are shown in Figure 2. In children’s venous blood, the medians for creatinine concentrations were 0.80 mg/dL (IQR, 0.71-0.93 mg/dL) at birth, 0.21 mg/dL (IQR, 0.18-0.25 mg/dL) at age 2 months, and 0.26 mg/dL (IQR, 0.24-0.29 mg/dL) at age 14 to 16 months. The medians for urea concentrations were 9.52 mg/dL (IQR, 7.84-11.20 mg/dL) at birth, 6.16 mg/dL (IQR, 4.90-7.56 mg/dL) at age 2 months, and 13.17 mg/dL (IQR, 10.64-15.13 mg/dL) at age 14 to 16 months.

### Reference Intervals for Creatinine and Urea in Children at Birth, Age 2 Months, and Age 14 to 16 Months

At the time of birth, GA was found to be associated with newborn venous blood concentrations of both creatinine (median [IQR], 0.77 [0.70-0.88] mg/dL for 37-39 weeks’ GA vs 0.84 [0.75-0.95] mg/dL for 40-42 weeks’ GA; *P* < .001) and urea (median [IQR], 9.10 [7.28-10.92] mg/dL for 37-39 weeks’ GA vs 10.08 [8.40-11.76] mg/dL for 40-42 weeks’ GA; *P* < .001). However, sex was not associated with concentrations of creatinine (median [IQR], 0.79 [0.71-0.92] mg/dL for female newborns vs 0.83 [0.72-0.98] mg/dL for male newborns; *P* = .06) or urea (median [IQR], 9.52 [7.28-10.92] mg/dL for female newborns vs 9.80 [7.84-11.76] mg/dL for male newborns; *P* = .07) (eTables 3-6 in the Supplement). Thus, reference intervals for newborns at the time of birth were stratified by GA (37-39 weeks and 40-42 weeks).

Reference intervals for creatinine and urea concentrations accounting for maternal comorbidity and outliers (model 4) are shown in Table 2 (with existing local reference intervals from Rigshospitalet provided for comparison). Reference intervals for creatinine concentrations were 0.54 to 1.08 mg/dL for newborns born at 37 to 39 weeks’ GA, 0.57 to 1.19 mg/dL for newborns born at 40 to 42 weeks’ GA, 0.13 to 0.31 mg/dL for infants aged 2 months, and 0.18 to 0.34 mg/dL for children aged 14 to 16 months. Reference intervals for urea concentrations were 5.32 to 14.67 mg/dL for for newborns born at 37 to 39 weeks’ GA, 5.60 to 14.85 mg/dL for at newborns born at 40 to 42 weeks’ GA, 3.08-10.08 mg/dL for infants aged 2 months, and 8.12-17.95 mg/dL for children aged 14 to 16 months.

**Table 2.** Reference Intervals for Creatinine and Urea Concentrations in Children’s Venous Blood

Venous blood concentration	Reference interval (No. of participants)			
	Newborn		Infant (age 2 mo)	Child (age 14-16 mo)
	GA 37-39 wk	GA 40-42 wk		
**Creatinine**				
New reference interval from current study, 2.5-97.5 percentile, mg/dL^a^	0.54-1.08 (144)	0.57-1.19 (162)	0.13-0.31 (268)	0.18-0.34 (115)
Existing local reference interval, mg/dL^b^	0.42-0.92 (5)^c^		0.19-0.46 (95)^d^	0.17-0.35 (45)^e^
**Urea**				
New reference interval from current study, 2.5-97.5 percentile, mg/dL^a^	5.32-14.67 (144)	5.60-14.85 (165)	3.08-10.08 (288)	8.12-17.95 (116)
Existing local reference interval, mg/dL^b^	3.92-15.10 (NR)^f^		5.04-15.10 (NR)^g^	

Abbreviations: GA, gestational age; NR, not reported.

SI conversion factors: To convert creatinine from milligrams per deciliter to micromoles per liter, multiply by 88.4; to convert urea from milligrams per deciliter to millimoles per liter, multiply by 0.357.

^a^ Reference intervals were adjusted for outliers and exclusion criteria and were derived from newborn venous blood at the time of birth (2016-2018), infant venous blood at age 2 months, and children’s venous blood at age 14 to 16 months (2016-2020). Outliers were adjusted as recommended by the *Tietz Textbook of Clinical Chemistry and Molecular Diagnostics*.^36^ Exclusion criteria were preeclampsia, maternal diabetes, maternal kidney disease, congenital kidney deformity, maternal body mass index (calculated as weight in kilograms divided by height in meters squared) greater than 35, and small for GA at birth. Newborn reference intervals from the time of birth were stratified by GA.

^b^ The existing local reference intervals were from the Clinical Biochemical Department of Copenhagen University Hospital–Rigshospitalet in Copenhagen, Denmark.

^c^ At age 0 to 1 day (determined from Boer et al^4^).

^d^ At age 14 days to 2 months (determined from Boer et al^4^).

^e^ At age 1 to 3 years (determined from Ceriotti et al^10^).

^f^ At age 0 to 2 months.

^g^ At age 2 months to 2 years.

Reference intervals for creatinine and urea concentrations for all 4 models are provided in eTable 7 and eTable 8 in the Supplement. Across the 4 models, the reference intervals for levels of creatinine and urea only varied slightly in both venous blood at birth, 2 months after birth, and 14 to 16 months after birth. The number of children with concentrations categorized as low or high using the presently generated and existing reference intervals are shown in eTable 9 in the Supplement. For creatinine, 26.4% of newborns assessed using the existing reference intervals would have been categorized as having high venous concentrations compared with 4.5% of newborns assessed using the reference intervals generated in this study. Reference intervals for umbilical blood are presented for creatinine in eTable 10 in the Supplement and for urea in eTable 11 in the Supplement.

### Maternal Comorbidity and Risk of High Concentrations of Creatinine and Urea in Umbilical Cord Blood

**Figure 3.**
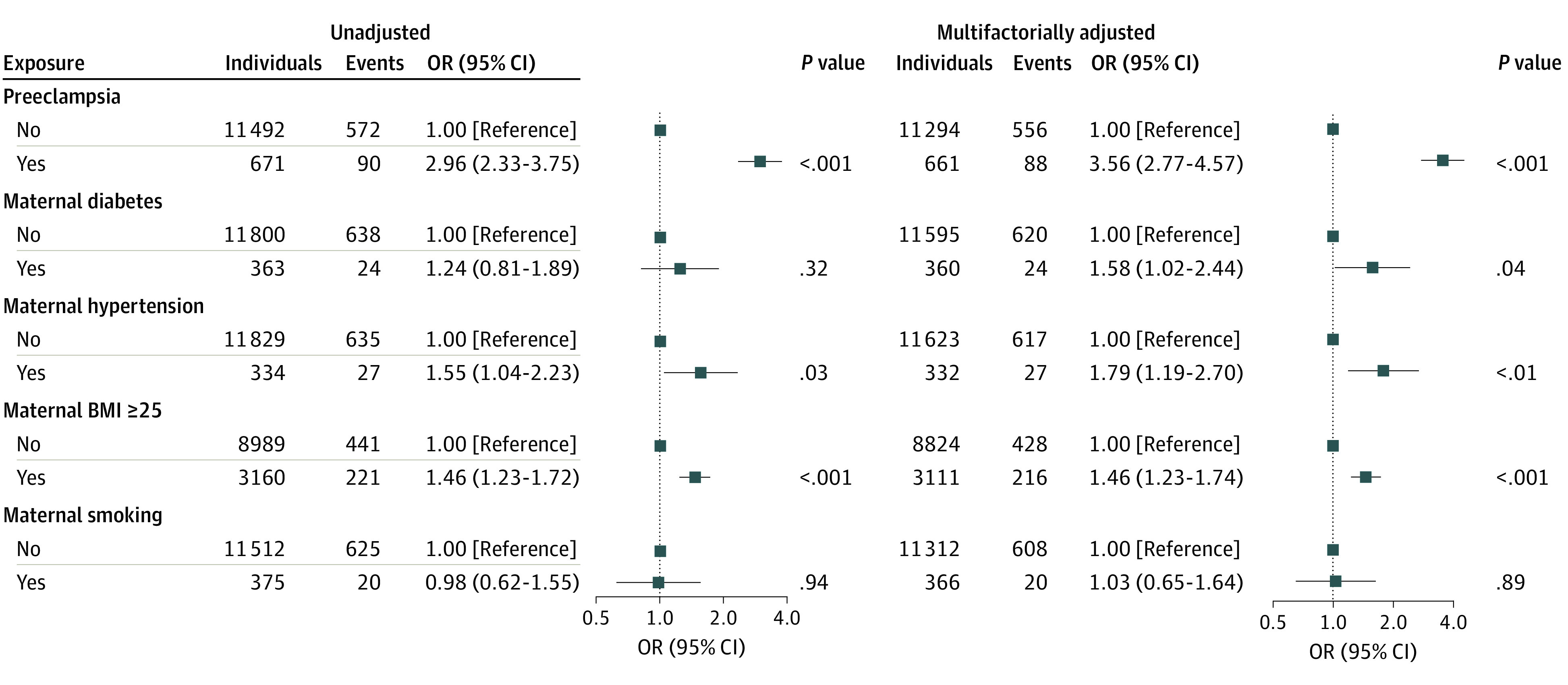
Association of Maternal Comorbidities With Risk of High Neonatal Creatinine Concentrations Odds ratios (ORs) of a newborn having an umbilical cord blood creatinine concentration higher than the upper reference limit (0.98 mg/dL) as a function of maternal comorbidities. The ORs, 95% CIs, and *P* values were derived from logistic regression analysis. Individuals represent the number of newborns with and without recorded maternal comorbidity. Events represent the number of newborns with a creatinine concentration higher than the upper reference limit. In the multifactorially adjusted model, ORs were adjusted for maternal age at birth, child’s sex, gestational age, birth weight, birth length, multiple births, and placental-fetal weight ratio higher or lower than the median. BMI indicates body mass index (calculated as weight in kilograms divided by height in meters squared).

To examine the association of maternal comorbidity with creatinine and urea concentrations in umbilical cord blood, odds ratios (ORs) were calculated using a logistic regression analysis with cutoffs corresponding to the upper reference interval limit (0.98 mg/dL for creatinine and 14.29 mg/dL for urea) (eTable 10 and eTable 11 in the Supplement). The models included umbilical cord blood samples from newborns of all GAs; however, a sensitivity analysis including only those born at 37 to 42 weeks’ GA yielded similar results. Multifactorially adjusted ORs for umbilical cord concentrations higher than the upper reference interval limit in newborns exposed to maternal preeclampsia were 3.56 (95% CI, 2.77-4.57) for creatinine and 3.91 (95% CI, 3.21-4.91) for urea. Corresponding adjusted ORs were 1.58 (95% CI, 1.02-2.44) for creatinine and 2.76 (95% CI, 1.97-3.86) for urea among newborns exposed to maternal diabetes, 1.79 (95% CI, 1.19-2.70) for creatinine and 2.21 (95% CI, 1.57-3.13) for urea among newborns exposed to maternal hypertension, 1.46 (95% CI, 1.23-1.74) for creatinine and 1.19 (95% CI, 1.00-1.41) for urea among newborns exposed to maternal overweight (defined as BMI ≥25), and 1.03 (95% CI, 0.65-1.64) for creatinine and 0.72 (95% CI, 0.43-1.19) for urea among newborns exposed to maternal smoking (Figure 3; eFigure 2 in the Supplement). The adjusted ORs for newborns exposed to maternal kidney disease were 5.04 (95% CI, 1.06-24.00) for creatinine and 3.55 (95% CI, 0.77-16.32) for urea.

### Maternal Comorbidities and Risk of High Concentrations of Creatinine and Urea in Children’s Venous Blood

Because maternal preeclampsia was the most significant exposure for high creatinine (OR, 3.56; 95% CI, 2.77-4.57) and urea (OR, 3.91; 95% CI, 3.21-4.91) concentrations in umbilical cord blood at birth (Figure 3; eFigure 2 in the Supplement), we also tested newborn venous blood. At birth, multifactorially adjusted ORs among newborns exposed to maternal preeclampsia were 9.40 (95% CI, 1.68-52.54) for the risk of a venous creatinine concentration higher than the upper reference limit, 4.29 (95% CI, 1.32-13.93) for the risk of a venous creatinine concentration higher than the 90th percentile, 3.10 (95% CI, 1.14-8.46) for the risk of a venous creatinine concentration higher than the 80th percentile, and 1.65 (95% CI, 0.63-4.27) for the risk of a venous creatinine concentration higher than the 50th percentile (eFigure 3 in the Supplement). Results remained significant when additionally adjusting for maternal creatinine concentrations at birth (higher than the upper reference limit: OR, 112.31; 95% CI, 4.18-3016.38; *P* = .005). Similar patterns were observed at age 2 months (>90th percentile: OR, 1.75; 95% CI, 0.46-6.63; *P* = .41) (eFigure 4 in the Supplement) and age 14 to 16 months (>80th percentile: OR, 2.58; 95% CI, 0.40-16.50; *P* = .32); however, the results were not statistically significant. Preeclampsia was found to be associated with a 3-fold increase in the risk of children’s urea concentrations higher than the 50th percentile at birth (OR, 3.44; 95% CI, 1.25-9.47; *P* = .02) (eFigure 5 in the Supplement). When adjusting for maternal urea concentration at birth, the association between preeclampsia and high newborn urea concentration was attenuated (higher than the upper reference limit: OR, 0.03 (95% CI, 0-11.44; *P* = .26). No associations between preeclampsia and urea concentrations were found at 2 months after birth (higher than the upper reference limit: OR, 1.98; 95% CI, 0.20-19.29; *P* = .56) (eFigure 6 in the Supplement) or 14 to 16 months after birth (higher than the 80th percentile: OR, 0.65; 95% CI, 0.08-5.52; *P* = .69).

## Discussion

The principal contribution of the present large cohort study was the generation of more robust and GA-dependent reference intervals for creatinine and urea in newborns. Furthermore, maternal preeclampsia was found to be associated with an increased risk of high newborn creatinine and urea concentrations.

In this study of umbilical and venous creatinine and urea concentrations in a large cohort of healthy term newborns with follow-up and parallel maternal venous samples, findings were consistent with previous studies reporting correlations of creatinine and urea in maternal venous blood with either fetal, umbilical cord, or newborn venous blood.^9,24,27,28,29^ Overall, creatinine and urea concentrations in umbilical and maternal blood were similar, whereas concentrations in newborn venous blood collected approximately 2 hours after birth differed significantly from those in umbilical and maternal blood, suggesting an accumulation after umbilical cord clamping. The equations provided in this study, however, enabled estimation of newborn creatinine and urea concentrations from umbilical cord blood and may thus prevent some venipuncture procedures in newborns.

Robust reference intervals are important for clinical decision-making; however, current Danish reference intervals of creatinine concentrations through the first year of life have been determined based on small groups of children (ranging from 5-95 children).^4,10,37^ In the present study, we were able to generate reference intervals based on healthy term infants, and we included a sufficient number of individuals to justify stratification by GA given that creatinine and urea concentrations differed significantly by GA. Stratification by GA was also supported by a recent review.^25^ In addition, newborns were excluded if their mothers had diagnoses that may have had implications for kidney function markers, which avoided confounding of the reference intervals by extreme values. Compared with previous studies,^7,11,12,13^ the presently generated reference intervals for creatinine concentrations at birth were based on a larger number of individuals and a more homogeneous population. A previous study,^10^ which included a similar number of children aged approximately 14 to 16 months and was comparable in size with our study, supported the present findings. In addition, the presently generated reference intervals categorized substantially fewer children with low or high creatinine and urea concentrations compared with existing Danish intervals and may thus provide a better basis for clinical decision-making when assessing term neonates. Furthermore, the reference intervals may help to identify children at risk of AKI. The reference intervals for urea found in the present study were narrower and applied to more well-defined age groups compared with current Danish reference intervals.

Notably, we found maternal comorbidity to be associated with the risk of high creatinine and urea concentrations in the newborn. Preeclampsia, maternal diabetes, maternal hypertension, and maternal overweight were associated with an increased risk of high creatinine and urea concentrations in the newborn, supporting previous observations on the risk of kidney disease associated with these comorbidities.^18,38,39,40^ Preeclampsia was associated with an up to 4-fold increased risk of high newborn creatinine (OR, 3.56) or urea (OR, 3.91) concentration in umbilical cord blood. This increased risk was sustained in newborn venous blood samples at birth, and a pattern was present at age 2 months, suggesting that attention should be given to kidney function in these children. This finding was supported by previous studies^18,41^ reporting pregnancy-induced hypertension to be associated with high creatinine concentrations and AKI in premature or hospitalized neonates. Because preeclampsia has been reported to be associated with high maternal creatinine concentrations,^42^ it is uncertain whether the risk of high newborn creatinine and urea concentrations associated with preeclampsia reflected higher maternal concentrations. Notably, we were able to adjust for maternal creatinine serum levels. The association between preeclampsia and high newborn creatinine concentrations persisted after this adjustment, suggesting that our findings could not solely be explained by maternal concentrations. More in-depth discussion is provided in the eDiscussion in the Supplement.

### Strengths and Limitations

This study has several strengths, including the large prospective population-based cohort of healthy term newborns, with 13 354 umbilical cord blood samples and parallel venous blood samples collected from 444 individuals at birth and follow-up conducted among 363 children at age 2 months and 156 children at age 14 to 16 months. This large sample combined with follow-up allowed us to generate reference intervals of kidney function markers in 3 age groups during the first 16 months of life in healthy children. Other major strengths are the paired samples from both maternal venous blood and umbilical cord and newborn venous blood at birth; the extensive information on diagnoses; and the high participation rate in the CBHS, with enrollment of 55% of infants born in the participating hospitals.^33^

The study also has limitations, including the overrepresentation of participants with higher socioeconomic status and educational level and mothers with Danish ethnicity.^33^ These limitations are, however, well-known issues in voluntary epidemiological studies and are, to some extent, compensated for by the high participation rate of the study. The maternal population had low BMI and a low incidence of comorbidities; however, these characteristics are representative of the entire population giving birth during the same period in the same hospitals.^33^ The number of newborn venous samples (n = 444) is small compared with the number of umbilical cord samples (n = 13 354); the venous samples were, however, collected from healthy newborns who did not otherwise require blood sampling. The equations for estimating newborn concentrations from umbilical blood provided in this study warrant validation in other cohorts of healthy term children. Moreover, the study mainly includes healthy term newborns; thus, it is not certain whether the results are generalizable to preterm newborns or newborns with illness. As suggested by the Tietz method,^36^ at least 120 individuals are recommended for creating reference intervals. This sample size was only just violated in the age 14- to 16-month group after exclusion of outliers and exclusion criteria due to less willingness to continue study participation as the children grew older. However, considerably more individuals are included in the presently generated reference intervals compared with existing intervals.

## Conclusions

This cohort study generated robust reference intervals for creatinine and urea concentrations stratified by GA at birth, age 2 months, and age 14 to 16 months and described the development of creatinine and urea concentrations in the first period of life. Furthermore, the study found an association between maternal comorbidities, especially preeclampsia, and an increased risk of high newborn creatinine and urea concentrations. This association suggests that more attention should be given to kidney function in children of mothers with preeclampsia. These novel findings may improve clinical decision-making and increase understanding of the consequences of maternal comorbidities for kidney function in newborns and infants.

## Notes

Supplement. **eMethods.** Biochemical Measurement of Creatinine and Urea and Covariates **eDiscussion.** Creatinine and Urea Concentrations **eTable 1.** Maternal and Newborn Diagnoses **eTable 2.** Assessment of Baseline Characteristics of Participants With and Without Missing Variables **eTable 3.** Creatinine Concentrations (mg/dL) Stratified by Gestational Age (GA) in Umbilical Cord Blood; Newborn Venous Blood at Birth, Age 2 Months, and Age 14-16 Months; and Maternal Venous Blood at Birth **eTable 4.** Urea Concentrations (mg/dL) Stratified by Gestational Age (GA) in Umbilical Cord Blood; Newborn Venous Blood at Birth, Age 2 Months, and Age 14-16 Months; and Maternal Venous Blood at Birth **eTable 5.** Creatinine Concentrations (mg/dL) Stratified by Sex in Umbilical Cord Blood; Newborn Venous Blood at Birth, Age 2 Months, and Age 14-16 Months; and Maternal Venous Blood at Birth **eTable 6.** Urea Concentrations (mg/dL) Stratified by Sex in Umbilical Cord Blood; Newborn Venous Blood at Birth, Age 2 Months, and Age 14-16 Months; and Maternal Venous Blood at Birth **eTable 7.** Reference Intervals for Creatinine Concentrations in Children’s Venous Blood **eTable 8.** Reference Intervals for Urea Concentrations in Children’s Venous Blood **eTable 9.** New and Existing Reference Intervals for Creatinine and Urea Concentrations and Number of Children From the COMPARE Study With Concentrations Higher and Lower Than the Reference Limits **eTable 10.** Reference Intervals for Creatinine Concentrations in Umbilical Cord Blood **eTable 11.** Reference Intervals for Urea Concentrations in Umbilical Cord Blood **eFigure 1.** Study Flowchart **eFigure 2.** Consequences of Maternal Comorbidities for the Risk of High Urea Concentrations in the Newborn **eFigure 3.** Consequences of Maternal Preeclampsia for the Risk of High Creatinine Concentrations in Newborn Venous Blood at Birth **eFigure 4.** Consequences of Maternal Preeclampsia for the Risk of High Creatinine Concentrations in Infant Venous Blood at 2 Months **eFigure 5.** Consequences of Maternal Preeclampsia for Urea Concentrations in Newborn Venous Blood at Birth **eFigure 6.** Consequences of Maternal Preeclampsia for Urea Concentrations in Infant Venous Blood at 2 Months
eReferences

## References

[1] Hoseini R, Otukesh H, Rahimzadeh N, Hoseini S. Glomerular function in neonates. Iran J Kidney Dis. 2012;6(3):166-172. 22555478

[2] Iacobelli S, Guignard JP. Maturation of glomerular filtration rate in neonates and infants: an overview. Pediatr Nephrol. 2021;36(6):1439-1446. doi:10.1007/s00467-020-04632-1 32529323

[3] Rhodin MM, Anderson BJ, Peters AM, . Human renal function maturation: a quantitative description using weight and postmenstrual age. Pediatr Nephrol. 2009;24(1):67-76. doi:10.1007/s00467-008-0997-5 18846389

[4] Boer DP, de Rijke YB, Hop WC, Cransberg K, Dorresteijn EM. Reference values for serum creatinine in children younger than 1 year of age. Pediatr Nephrol. 2010;25(10):2107-2113. doi:10.1007/s00467-010-1533-y 20505955 PMC2923720

[5] Bueva A, Guignard JP. Renal function in preterm neonates. Pediatr Res. 1994;36(5):572-577. doi:10.1203/00006450-199411000-00005 7877873

[6] Feldman H, Guignard JP. Plasma creatinine in the first month of life. Arch Dis Child. 1982;57(2):123-126. doi:10.1136/adc.57.2.123 7065707 PMC1627513

[7] Miall LS, Henderson MJ, Turner AJ, . Plasma creatinine rises dramatically in the first 48 hours of life in preterm infants. Pediatrics. 1999;104(6):e76. doi:10.1542/peds.104.6.e76 10586010

[8] Gallini F, Maggio L, Romagnoli C, Marrocco G, Tortorolo G. Progression of renal function in preterm neonates with gestational age < or = 32 weeks. Pediatr Nephrol. 2000;15(1-2):119-124. doi:10.1007/s004670000356 11095027

[9] Filler G, Lopes L, Harrold J, Bariciak E. β-Trace protein may be a more suitable marker of neonatal renal function. Clin Nephrol. 2014;81(4):269-276. doi:10.5414/CN108089 24548934

[10] Ceriotti F, Boyd JC, Klein G, ; IFCC Committee on Reference Intervals and Decision Limits (C-RIDL). Reference intervals for serum creatinine concentrations: assessment of available data for global application. Clin Chem. 2008;54(3):559-566. doi:10.1373/clinchem.2007.099648 18202155

[11] Bariciak E, Yasin A, Harrold J, Walker M, Lepage N, Filler G. Preliminary reference intervals for cystatin C and beta-trace protein in preterm and term neonates. Clin Biochem. 2011;44(13):1156-1159. doi:10.1016/j.clinbiochem.2011.06.987 21771588

[12] Schwartz GJ, Feld LG, Langford DJ. A simple estimate of glomerular filtration rate in full-term infants during the first year of life. J Pediatr. 1984;104(6):849-854. doi:10.1016/S0022-3476(84)80479-5 6726515

[13] Rudd PT, Hughes EA, Placzek MM, Hodes DT. Reference ranges for plasma creatinine during the first month of life. Arch Dis Child. 1983;58(3):212-215. doi:10.1136/adc.58.3.212 6838252 PMC1627816

[14] Treiber M, Pečovnik Balon B, Gorenjak M. A new serum cystatin C formula for estimating glomerular filtration rate in newborns. Pediatr Nephrol. 2015;30(8):1297-1305. doi:10.1007/s00467-014-3029-7 25956698

[15] Wilhelm-Bals A, Combescure C, Chehade H, Daali Y, Parvex P. Variables of interest to predict glomerular filtration rate in preterm newborns in the first days of life. Pediatr Nephrol. 2020;35(4):703-712. doi:10.1007/s00467-019-04257-z 31001662

[16] van Donge T, Allegaert K, Gotta V, . Characterizing dynamics of serum creatinine and creatinine clearance in extremely low birth weight neonates during the first 6 weeks of life. Pediatr Nephrol. 2021;36(3):649-659. doi:10.1007/s00467-020-04749-3 32944826 PMC7851041

[17] Pottel H, Vrydags N, Mahieu B, Vandewynckele E, Croes K, Martens F. Establishing age/sex related serum creatinine reference intervals from hospital laboratory data based on different statistical methods. Clin Chim Acta. 2008;396(1-2):49-55. doi:10.1016/j.cca.2008.06.017 18621041

[18] Iacobelli S, Bonsante F, Ferdinus C, Labenne M, Gouyon JB. Factors affecting postnatal changes in serum creatinine in preterm infants with gestational age <32 weeks. J Perinatol. 2009;29(3):232-236. doi:10.1038/jp.2008.203 19078973

[19] Gordjani N, Burghard R, Leititis JU, Brandis M. Serum creatinine and creatinine clearance in healthy neonates and prematures during the first 10 days of life. Eur J Pediatr. 1988;148(2):143-145. doi:10.1007/BF00445923 3234436

[20] De Curtis M, Rigo J. Nutrition and kidney in preterm infant. J Matern Fetal Neonatal Med. 2012;25(suppl 1):55-59. doi:10.3109/14767058.2012.663167 22394021

[21] GBD 2019 Risk Factors Collaborators. Global burden of 87 risk factors in 204 countries and territories, 1990-2019: a systematic analysis for the Global Burden of Disease study 2019. Lancet. 2020;396(10258):1223-1249. doi:10.1016/S0140-6736(20)30752-2 33069327 PMC7566194

[22] Dolea C, AbouZahr C. Global burden of hypertensive disorders of pregnancy in the year 2000. Evidence and Information for Policy (EIP). World Health Organization; 2003. Accessed November 25, 2021. https://www.researchgate.net/profile/Carla-Abouzahr/publication/265222498_Global_Burden_of_Disease_2000_Global_burden_of_hypertensive_disorders_of_pregnancy_in_the_year_2000/links/54ac0eaa0cf25c4c472fe1a7/Global-Burden-of-Disease-2000-Global-burden-of-hypertensive-disorders-of-pregnancy-in-the-year-2000.pdf

[23] Filler G, Kirpalani A, Urquhart B. Handling of drugs in children with abnormal renal function. In: Avner E, Harmon W, Niaudet P, Yoshikawa N, Emma F, Goldstein S, eds. *Pediatric Nephrology*. Springer; 2016:2267-2293.

[24] Guignard JP, Drukker A. Why do newborn infants have a high plasma creatinine? Pediatrics. 1999;103(4):e49. doi:10.1542/peds.103.4.e49 10103341

[25] Filler G, Bhayana V, Schott C, Díaz-González de Ferris ME. How should we assess renal function in neonates and infants? Acta Paediatr. 2021;110(3):773-780. doi:10.1111/apa.15557 32869283

[26] Davis BM, Miller RK, Brent RL, Koszalka TR. Materno-fetal transport of creatine in the rat. Biol Neonate. 1978;33(1-2):43-54. doi:10.1159/000241050 656521

[27] Lao TT, Loong EP, Chin RK, Lam YM. Renal function in the newborn: newborn creatinine related to birth weight, maturity and maternal creatinine. Gynecol Obstet Invest. 1989;28(2):70-72. doi:10.1159/000293517 2792916

[28] Nava S, Bocconi L, Zuliani G, Kustermann A, Nicolini U. Aspects of fetal physiology from 18 to 37 weeks’ gestation as assessed by blood sampling. Obstet Gynecol. 1996;87(6):975-980. doi:10.1016/0029-7844(96)00056-7 8649709

[29] Manzke H, Spreter von Kreudenstein P, Dörner K, Kruse K. Quantitative measurements of the urinary excretion of creatinine, uric acid, hypoxanthine and xanthine, uracil, cyclic AMP, and cyclic GMP in healthy newborn infants. Eur J Pediatr. 1980;133(2):157-161. doi:10.1007/BF00441585 6244960

[30] Lee SY, Moon JE, Park SH. Longitudinal changes in serum creatinine levels and urinary biomarkers in late preterm infants during the first postnatal week: association with acute kidney injury and treatment with aminoglycoside. Children (Basel). 2021;8(10):896. doi:10.3390/children8100896 34682161 PMC8534773

[31] World Medical Association. World Medical Association Declaration of Helsinki: ethical principles for medical research involving human subjects. *JAMA*. 2013;310(20):2191-2194. doi:10.1001/jama.2013.281053 24141714

[32] Sillesen AS, Raja AA, Pihl C, . Copenhagen Baby Heart Study: a population study of newborns with prenatal inclusion. Eur J Epidemiol. 2019;34(1):79-90. doi:10.1007/s10654-018-0448-y 30306423

[33] Vøgg ROB, Basit S, Raja AA, . Cohort profile: the Copenhagen Baby Heart Study (CBHS). Int J Epidemiol. 2022;50(6):1778-1779. doi:10.1093/ije/dyab147 34999847

[34] Nielsen ST, Strandkjær N, Juul Rasmussen I, . Coagulation parameters in the newborn and infant—the Copenhagen Baby Heart and COMPARE studies. Clin Chem Lab Med. 2021;60(2):261-270. doi:10.1515/cclm-2021-0967 34752018

[35] Marsál K, Persson PH, Larsen T, Lilja H, Selbing A, Sultan B. Intrauterine growth curves based on ultrasonically estimated foetal weights. Acta Paediatr. 1996;85(7):843-848. doi:10.1111/j.1651-2227.1996.tb14164.x 8819552

[36] Burtis CA, Ashwood ER, Bruns DE. *Tietz Textbook of Clinical Chemistry and Molecular Diagnostics*. 5th ed. W.B. Saunders Company; 2012.

[37] Rigshospitalets Labportal. Updated October 12, 2022. Accessed November 25, 2021. https://labportal.rh.dk/

[38] Nelson RG, Morgenstern H, Bennett PH. Intrauterine diabetes exposure and the risk of renal disease in diabetic Pima Indians. Diabetes. 1998;47(9):1489-1493. doi:10.2337/diabetes.47.9.1489 9726239

[39] Luyckx VA, Brenner BM. Birth weight, malnutrition and kidney-associated outcomes—a global concern. Nat Rev Nephrol. 2015;11(3):135-149. doi:10.1038/nrneph.2014.251 25599618

[40] Hsu CW, Yamamoto KT, Henry RK, De Roos AJ, Flynn JT. Prenatal risk factors for childhood CKD. J Am Soc Nephrol. 2014;25(9):2105-2111. doi:10.1681/ASN.2013060582 24744441 PMC4147970

[41] Ghobrial EE, Elhouchi SZ, Eltatawy SS, Beshara LO. Risk factors associated with acute kidney injury in newborns. *Saudi J Kidney Dis Transpl*. 2018;29(1):81-87. doi:10.4103/1319-2442.225179 29456211

[42] Jhee JH, Lee S, Park Y, . Prediction model development of late-onset preeclampsia using machine learning–based methods. PLoS One. 2019;14(8):e0221202. doi:10.1371/journal.pone.0221202 31442238 PMC6707607

