# Supplementary material for: Markers of Kidney Function in Early Childhood and Association With Maternal Comorbidity
Source: JAMA Netw Open. 2022 Nov 21;5(11):e2243146. doi: 10.1001/jamanetworkopen.2022.43146 (PMC9679880; doi:10.1001/jamanetworkopen.2022.43146)
Supplement: Supplement. — eMethods. Biochemical Measurement of Creatinine and Urea and Covariates eDiscussion. Creatinine and Urea Concentrations eTable 1. Maternal and Newborn Diagnoses eTable 2. Assessment of Baseline Characteristics of Participants With and Without Missing Variables eTable 3. Creatinine Concentrations (mg/dL) Stratified by Gestational Age (GA) in Umbilical Cord Blood; Newborn Venous Blood at Birth, Age 2 Months, and Age 14-16 Months; and Maternal Venous Blood at Birth eTable 4. Urea Concentrations (mg/dL) Stratified by Gestational Age (GA) in Umbilical Cord Blood; Newborn Venous Blood at Birth, Age 2 Months, and Age 14-16 Months; and Maternal Venous Blood at Birth eTable 5. Creatinine Concentrations (mg/dL) Stratified by Sex in Umbilical Cord Blood; Newborn Venous Blood at Birth, Age 2 Months, and Age 14-16 Months; and Maternal Venous Blood at Birth eTable 6. Urea Concentrations (mg/dL) Stratified by Sex in Umbilical Cord Blood; Newborn Venous Blood at Birth, Age 2 Months, and Age 14-16 Months; and Maternal Venous Blood at Birth eTable 7. Reference Intervals for Creatinine Concentrations in Children’s Venous Blood eTable 8. Reference Intervals for Urea Concentrations in Children’s Venous Blood eTable 9. New and Existing Reference Intervals for Creatinine and Urea Concentrations and Number of Children From the COMPARE Study With Concentrations Higher and Lower Than the Reference Limits eTable 10. Reference Intervals for Creatinine Concentrations in Umbilical Cord Blood eTable 11. Reference Intervals for Urea Concentrations in Umbilical Cord Blood eFigure 1. Study Flowchart eFigure 2. Consequences of Maternal Comorbidities for the Risk of High Urea Concentrations in the Newborn eFigure 3. Consequences of Maternal Preeclampsia for the Risk of High Creatinine Concentrations in Newborn Venous Blood at Birth eFigure 4. Consequences of Maternal Preeclampsia for the Risk of High Creatinine Concentrations in Infant Venous Blood at 2 Months eFigure 5. Consequences of Maternal Preec [file jamanetwopen-e2243146-s001.pdf]

Supplemental Online Content

Mohr Lytsen R, Taageby Nielsen S, Kongsgaard Hansen M, et al. Markers of kidney function in early childhood and association with maternal comorbidity. *JAMA Netw Open*. 2022;5(11):e2243146. doi:10.1001/jamanetworkopen.2022.43146

eMethods. Biochemical Measurement of Creatinine and Urea and Covariates

eDiscussion. Creatinine and Urea Concentrations

eTable 1. Maternal and Newborn Diagnoses

eTable 2. Assessment of Baseline Characteristics of Participants With and Without Missing Variables

eTable 3. Creatinine Concentrations (mg/dL) Stratified by Gestational Age (GA) in Umbilical Cord Blood; Newborn Venous Blood at Birth, Age 2 Months, and Age 14-16 Months; and Maternal Venous Blood at Birth

eTable 4. Urea Concentrations (mg/dL) Stratified by Gestational Age (GA) in Umbilical Cord Blood; Newborn Venous Blood at Birth, Age 2 Months, and Age 14-16 Months; and Maternal Venous Blood at Birth

eTable 5. Creatinine Concentrations (mg/dL) Stratified by Sex in Umbilical Cord Blood; Newborn Venous Blood at Birth, Age 2 Months, and Age 14-16 Months; and Maternal Venous Blood at Birth

eTable 6. Urea Concentrations (mg/dL) Stratified by Sex in Umbilical Cord Blood; Newborn Venous Blood at Birth, Age 2 Months, and Age 14-16 Months; and Maternal Venous Blood at Birth

eTable 7. Reference Intervals for Creatinine Concentrations in Children's Venous Blood

eTable 8. Reference Intervals for Urea Concentrations in Children's Venous Blood

eTable 9. New and Existing Reference Intervals for Creatinine and Urea Concentrations and Number of Children From the COMPARE Study With Concentrations Higher and Lower Than the Reference Limits

eTable 10. Reference Intervals for Creatinine Concentrations in Umbilical Cord Blood

eTable 11. Reference Intervals for Urea Concentrations in Umbilical Cord Blood

eFigure 1. Study Flowchart

eFigure 2. Consequences of Maternal Comorbidities for the Risk of High Urea Concentrations in the Newborn

eFigure 3. Consequences of Maternal Preeclampsia for the Risk of High Creatinine Concentrations in Newborn Venous Blood at Birth

eFigure 4. Consequences of Maternal Preeclampsia for the Risk of High Creatinine Concentrations in Infant Venous Blood at 2 Months

eFigure 5. Consequences of Maternal Preeclampsia for Urea Concentrations in Newborn Venous Blood at Birth

eFigure 6. Consequences of Maternal Preeclampsia for Urea Concentrations in Infant Venous Blood at 2 Months

eReferences

This supplemental material has been provided by the authors to give readers additional information about their work.

© 2022 Mohr Lytsen RM et al. *JAMA Network Open*.

eMethods. Biochemical Measurement of Creatinine and Urea and Covariates

Biochemical measurement of creatinine and urea

The umbilical blood and venous blood samples were analyzed for biochemical markers, including creatinine and urea, on standard hospital clinical biochemistry equipment [1]. The blood samples were collected in serum vacutainers [2], and serum creatinine (µmol/L) and urea (mmol/L) concentrations were measured by the Cobas 6000, c501 module, using the CREAJ Gen.2, Cobas c, Integra and the UREAL, Cobas c, Integra reagents for creatinine and urea (Roche, Basel, Switzerland). Creatinine in µmol/L and urea in mmol/L were converted to conventional units, mg/dL.

Covariates

Demographics and data on newborns and mothers were extracted from the local obstetric and Astraia fetal medicine databases. Gestational age (GA) was determined from obstetric ultrasonography and given in weeks. Maternal age was registered at delivery. Maternal body mass index (BMI) was self-reported and calculated as weight before pregnancy in kilograms divided by height in meters squared. Birth weight and length were measured at birth. Neonatal Appearance, Pulse, Grimace, Activity and Respiration (APGAR) score was assessed one and five minutes after birth using a score from 1-10, 7 to 10 considered reassuring. Neonates small for gestational age (SGA) were identified by the formula presented by Marsal et al. [3]. SGA was defined as a birth weight below the 2.3th percentile equivalent to a birth weight less than -22% of the average birth weight for a given gestational age. Placental weight was in grams (g). Placental-fetal weight ratio was determined as placental weight divided by birth weight and given in g/1g. Maternal and newborn diagnoses were registered using the International Classification of Diseases, Tenth Revision (ICD-10) codes. The following relevant morbidities are detailed in eTable 1: Maternal kidney disease, preeclampsia, maternal hypertension, maternal diabetes, placental insufficiency, and congenital malformation of the urinary system. Information on smoking is self-reported during pre-pregnancy interview with midwife in the first trimester. Umbilical cord blood refers to samples drawn from the umbilical vein after birth. Newborn venous blood and maternal venous blood refers to venous blood samples taken approximately 2 hours after birth. Child venous blood refers to blood samples taken at follow-up at 2 and 14-16 months, specified when used.

© 2022 Mohr Lytsen RM et al. *JAMA Network Open*.

eDiscussion. Creatinine and Urea Concentrations

Serum creatinine and urea concentrations are frequently used to estimate GFR in neonates. Creatinine and urea pass the placenta passively from mother to fetus by simple diffusion reaching an equilibrium between fetal and maternal concentrations[4-7]. This is supported by the presently observed high correlations between creatinine and urea concentrations in umbilical cord and maternal blood. Newborn concentrations have been suggested to reflect maternal concentrations for up to 72 hours[8,9], with a gradual increase during the first days of life[10-12]. In the present study, we do, however, observe the increase to occur within the first two hours after birth, suggesting newborn accumulation of creatinine after severing the umbilical cord. The exact mechanism underlying this accumulation is not certain, however, tubular reabsorption of creatinine across immature leaky tubules as well as dehydration may play a role[13,14]. Creatinine concentrations have been reported to start declining after the first days and the following year after birth[15,16], which is also found in in this study, thus this result is confirmatory. Urea is a poor single parameter for renal function. The presently observed decrease at two months and later increase at 14-16 months is likely reflecting the dietary protein intake being low on breast milk and increased on toddlers' diet[7,10,17].

© 2022 Mohr Lytsen RM et al. *JAMA Network Open*.

eTable 1. Maternal and Newborn Diagnoses

Diagnoses	ICD-10 codes	Description
Maternal kidney disease		
	N18, N189	Chronic kidney disease
	N039	Chronic glomerulonephritis
	N03	Chronic nephritic syndrome
	N131, N133	Hydronephrosis
	N139	Obstructive and reflux uropathy
	N289	Disorder of kidney and ureter incl. nephropathy
	Q600	unilateral renal agenesis
	Q613	polycystic kidney
	Q619	cystic kidney disease
	Q638, Q649	congenital malformations of the kidney or urinary system
Preeclampsia<br><br>Defined as hypertension after 20 weeks of gestation with a systolic blood pressure ≥140 and/or diastolic blood pressure ≥90 mmHg and proteinuria and/or sign of maternal organ dysfunction.		
	O100	Pre-existing essential hypertension complicating pregnancy, childbirth and puerperium
	O120	Gestational edema and proteinuria without hypertension
	O121	Gestational proteinuria
	O139	Gestational hypertension
	O140	Mild to moderate preeclampsia
	O141	Severe preeclampsia
	O142	HELLP (H=hemolysis; EL=Elevated Liver enzymes; LP=Low platelets) syndrome
	O159	Eclampsia
Maternal hypertension		
	O100	Pre-existing essential hypertension complicating pregnancy, childbirth, and puerperium
	O139	Gestational hypertension
Maternal diabetes		
	O240	preexisting type 1 diabetes mellitus
	O241	preexisting type 2 diabetes mellitus
	O244D	gestational diabetes
	O244E	gestational diabetes treated with insulin

© 2022 Mohr Lytsen RM et al. *JAMA Network Open*.

	O245	newly discovered manifest diabetes during pregnancy
**Diagnoses**	**ICD-10 codes**	**Description**
Placental insufficiency		
	O365	Maternal care for known or suspected poor fetal growth and placental insufficiency
	O438E	Placental dysfunction/infarction, subchorionic hematoma
	O431, O431E	Malformation of placenta (abnormal placenta nitric oxide synthase (NOS), circumvallate placenta) including vasa previa
Congenital malformation of the urinary system		
	Q600	Unilateral renal agenesis
	Q613	Polycystic kidney, unspecified
	Q619	Cystic kidney disease, unspecified
	Q620A/B	Congenital unilateral or bilateral hydronephrosis
	Q639	Congenital malformation of the kidney, unspecified
	Q649	Congenital malformation of the urinary system, unspecified

Registered ICD-10 (International Classification of Diseases, Tenth Revision) codes for maternal and newborn diagnoses used in the study.

© 2022 Mohr Lytsen RM et al. *JAMA Network Open*.

eTable 2. Assessment of Baseline Characteristics of Participants With and Without Missing Variables

	Females n=6,557 (49%)			Males n=6,797 (51%)		
**The Copenhagen Baby Heart Study (N=13,354)**	≥ 1 missing variable n=871 (13.3%)	Complete cases n=5,686 (86.7%)	P-values	≥ 1 missing variable n=840 (12.4%)	Complete cases n=5,957 (87.6%)	P-values
Gestational age, median (IQR), days	282 (275 – 287)	282 (274 – 287)	0.61	282 (274 – 288)	281 (273 – 287)	0.32
Maternal Age, median (IQR), years	31 (29 – 35)	31 (29 – 35)	0.80	32 (29 – 35)	31 (29 – 35)	0.88
Birth Weight, median (IQR), g	3456 (3138 – 3769)	3475 (3178 – 3779)	0.18	3574 (3275 – 3875)	3599 (3280 – 3915)	0.13
Birth Length, median (IQR), cm	51 (50 – 53)	51 (50 – 53)	0.32	52 (51 – 53)	52 (51 – 54)	0.59
Apgar Score, median (IQR), 5 min	10 (10 – 10)	10 (10 – 10)	0.14	10 (10 – 10)	10 (10 – 10)	<0.001
Small for Gestational Age, No. (%)	38 (4.4)	171 (3.0)	0.03	28 (3.3)	174 (2.9)	0.51
Placental weight, median (IQR), g	615 (530 – 713)	630 (543 – 720)	0.10	630 (540 – 740)	642 (554 – 740)	0.05
Placental-fetal weight ratio, median (IQR), g placenta / 1 g child	0.18 (0.16 – 0.20)	0.18 (0.16 – 0.20)	0.28	0.18 (0.16 – 0.20)	0.18 (0.16 – 0.20)	0.14
Placental insufficiency, No. (%)	46 (5.3)	331 (5.8)	0.54	45 (5.4)	268 (4.5)	0.26
Congenital malformation of the urinary system, No. (%)	0 (0.0)	6 (0.1)	0.34	4 (0.5)	12 (0.2)	0.11
Maternal BMI, median (IQR), kg/m^2	22 (21 – 25)	22 (21 – 25)	0.80	22 (20 – 25)	22 (21 – 25)	0.11
Preeclampsia, No. (%)	47 (5.4)	315 (5.5)	0.88	40 (4.8)	329 (5.5)	0.37
Maternal diabetes, No. (%)	21 (2.4)	153 (2.6)	0.64	23 (2.7)	193 (3.2)	0.45
Maternal Kidney Disease, No. (%)	3 (0.3)	4 (0.1)	0.02	0	7 (0.1)	0.32
Maternal Hypertension, No. (%)	22 (2.5)	159 (2.8)	0.66	25 (3.0)	150 (2.7)	0.62
Maternal Smoking, No. (%)	21 (2.8)	180 (3.2)	0.58	24 (3.4)	184 (3.1)	0.65
	Females n=217 (49%)			Males n=227 (51%)		
**The COMPARE study**	≥ 1 missing variable n=30 (13.8%)	Complete cases n=187 (86.2%)		≥ 1 missing variable n=39 (17.2%)	Complete cases n=188 (82.8%)	
Gestational age, median (IQR), days	276 (272 – 288)	280 (273 – 287)	0.44	280 (271 – 288)	281 (272 – 288)	0.96
Maternal Age, median (IQR), years	32 (29-37)	32 (29-36)	0.93	32 (28-35)	32 (29 – 35)	0.80
Birth Weight, median (IQR), g	3415 (3049 – 3557)	3430 (3168 – 3692)	0.44	3549 (3362 – 3891)	3528 (3337 – 3895)	0.96
Birth Length, median (IQR), cm	51 (50 – 52)	51 (50 – 53)	0.74	52 (50 – 54)	52 (51 – 54)	0.59
Apgar Score, median (IQR), 5 min	10 (10 – 10)	10 (10 – 10)	0.74	10 (10 – 10)	10 (10 – 10)	0.20
Small for Gestational Age, No. (%)	2 (6.7)	5 (2.7)	0.25	2 (5.1)	3 (1.6)	0.17
Placental weight, median (IQR), g	650 (577 – 760)	618 (550 – 710)	0.36	625 (578 – 785)	663 (569 – 771)	0.66

© 2022 Mohr Lytsen RM et al. *JAMA Network Open*.

Placental-fetal weight ratio, median (IQR), g placenta / 1 g child	0.19 (0.16 – 0.22)	0.19 (0.16 – 0.20)	0.37	0.18 (0.16 – 0.20)	0.19 (0.16 – 0.21)	0.61
Placental insufficiency, No. (%)	1 (3.3)	10 (5.3)	0.64	3 (7.7)	5 (2.7)	0.12
Congenital malformation of the urinary system, No. (%)	0	0		0	0	
Maternal BMI, median (IQR), kg/m^2	22 (20 – 25)	22 (21 – 25)	0.55	24 (21 – 27)	23 (20 – 25)	0.22
Preeclampsia, No. (%)	0	12 (6.4)	0.15	5 (12.8)	10 (5.3)	0.09
Maternal diabetes, No. (%)	1 (3.3)	2 (1.1) p=0.324	0.32	2 (5.1)	9 (4.8)	0.93
Maternal kidney disease, No. (%)	0	0		0	0	
Maternal hypertension, No. (%)	0	8 (4.3)	0.25	3 (7.7)	9 (4.8)	0.46
Maternal smoking, No. (%)	0	2 (1.1)	0.77	0	1 (0.5)	0.70

Baseline Characteristics in The Copenhagen Baby Heart Study and COMPARE study stratified by sex and complete cases and cases with more than 1 variable missing. Categorical variables are presented as numbers (percentages) and continuous variables as median (interquartile ranges). The variables are from the day of birth (2016-2018). Numbers may vary from flowchart due to missing variables. Gestational age (GA) was determined from obstetric ultrasonography and given in weeks. Maternal age was registered at delivery and given in years. Birth weight in grams and length in centimeters were measured at birth. Neonatal Appearance, Pulse, Grimace, Activity and Respiration (APGAR) score was assessed one and five minutes after birth using a score from 1-10, 7 to 10 considered reassuring. Small for gestational age was defined as a birth weight below the 2.3. percentile equivalent to a birth weight less than -22% of the average birth weight for a given gestational age [3]. Placental weight was measured in grams. Placental-fetal weight ratio was determined as placental weight divided by birth weight and given in g/1g. Maternal body mass index (BMI) was calculated as self-reported weight before pregnancy in kilograms divided by height in meters squared. Placental insufficiency, congenital malformation of the urinary system, preeclampsia, maternal diabetes, maternal kidney disease and maternal hypertension are detailed in eTable 1 in the Supplementary. Information on smoking is self-reported during pre-pregnancy interview with midwife in the first trimester. P-values by Mann-Whitney U test or Pearson's χ^2-test.

© 2022 Mohr Lytsen RM et al. *JAMA Network Open*.

eTable 3. Creatinine Concentrations (mg/dL) Stratified by Gestational Age (GA) in Umbilical Cord Blood; Newborn Venous Blood at Birth, Age 2 Months, and Age 14-16 Months; and Maternal Venous Blood at Birth

	GA 37 – 39	GA 40 - 42	P-value
**COMPARE - Creatinine concentration (mg/dL)**			
Umbilical cord blood	0.64 (0.55 – 0.74) (n=153)	0.70 (0.63 – 0.80) (n=167)	<0.001
Newborn venous blood (Birth)	0.77 (0.70 – 0.88) (n=171)	0.84 (0.75 – 0.95) (n=182)	<0.001
Infant venous blood (Two months)	0.21 (0.19 – 0.25) (n=144)	0.21 (0.18 – 0.24) (n=169)	0.20
Child venous blood (14-16 months)	0.27 (0.24 – 0.29) (n=63)	0.26 (0.21 – 0.29) (n=76)	0.18
Maternal venous blood (Birth)	0.63 (0.54 – 0.72) (n=210)	0.66 (0.59 – 0.76) (n=231)	0.01
**Copenhagen Baby Heart - Creatinine concentration (mg/dL)**			
Umbilical cord blood	0.67 (0.59 – 0.77) (n=4,814)	0.71 (0.63 – 0.81) (n=6,980)	<0.001

Creatinine concentrations are stratified by gestational age and presented as median (interquartile range) and n: number of individuals. P-values by Mann-Whitney U test. To convert mg/dL to µmol/L multiply by 88.4.

© 2022 Mohr Lytsen RM et al. *JAMA Network Open*.

eTable 4. Urea Concentrations (mg/dL) Stratified by Gestational Age (GA) in Umbilical Cord Blood; Newborn Venous Blood at Birth, Age 2 Months, and Age 14-16 Months; and Maternal Venous Blood at Birth

	GA 37 – 39	GA 40 - 42	P-value
**COMPARE - Urea concentration (mg/dL)**			
Umbilical cord blood	8.40 (7.28 – 10.36) (n=154)	9.80 (7.84 – 11.20) (n=171)	0.003
Newborn venous blood (Birth)	9.10 (7.28 – 10.92) (n=172)	10.08 (8.40 – 11.76) (n=185)	<0.001
Infant venous blood (Two months)	6.16 (4.76 – 7.56) (n=152)	6.16 (5.04 – 7.56) (n=176)	0.85
Child venous blood (14-16 months)	14.01 (11.48 – 16.24) (n=63)	12.89 (10.64 – 14.85) (n=78)	0.02
Maternal venous blood (Birth)	7.70 (6.16 – 9.52) (n=210)	8.40 (6.72 – 10.08) (n=230)	0.02
**Copenhagen Baby Heart - Urea concentration (mg/dL)**			
Umbilical cord blood	8.96 (7.28 – 10.92) (n=5,017)	9.52 (7.84 – 11.20) (n=7,282)	<0.001

Urea concentrations are stratified by gestational age and presented as median (interquartile range) and n: number of individuals. P-values by Mann-Whitney U test. To convert mg/dL to mmol/L multiply by 0.357.

© 2022 Mohr Lytsen RM et al. *JAMA Network Open*.

eTable 5. Creatinine Concentrations (mg/dL) Stratified by Sex in Umbilical Cord Blood; Newborn Venous Blood at Birth, Age 2 Months, and Age 14-16 Months; and Maternal Venous Blood at Birth

	Females	Males	P-value
**COMPARE - Creatinine concentration (mg/dL)**			
Umbilical cord blood	0.68 (0.60 – 0.75) (n=158)	0.68 (0.58 – 0.79) (n=162)	0.70
Newborn venous blood (Birth)	0.79 (0.71 – 0.92) (n=172)	0.83 (0.72 – 0.98) (n=181)	0.06
Infant venous blood (Two months)	0.21 (0.18 – 0.24) (n=142)	0.21 (0.18 – 0.25) (n=171)	0.76
Child venous blood (14-16 months)	0.26 (0.24 – 0.29) (n=67)	0.26 (0.23 – 0.29) (n=72)	0.75
Maternal venous blood (Birth)	0.64 (0.58 – 0.72) (n=215)	0.64 (0.57 – 0.75) (n=226)	0.89
**Copenhagen Baby Heart - Creatinine concentration (mg/dL)**			
Umbilical cord blood	0.69 (0.60 – 0.78) (n=5,954)	0.71 (0.61 – 0.80) (n=6,215)	<0.001

Creatinine concentrations are stratified by sex and presented as median (interquartile range) and n: number of individuals. P-values by Mann-Whitney U test. To convert mg/dL to µmol/L multiply by 88.4.

© 2022 Mohr Lytsen RM et al. *JAMA Network Open*.

eTable 6. Urea Concentrations (mg/dL) Stratified by Sex in Umbilical Cord Blood; Newborn Venous Blood at Birth, Age 2 Months, and Age 14-16 Months; and Maternal Venous Blood at Birth

	Females	Males	P-value
**COMPARE - Urea concentration (mg/dL)**			
Umbilical cord blood	9.10 (7.56 – 10.64) (n=162)	8.96 (7.56 – 11.48) (n=163)	0.33
Newborn venous blood (Birth)	9.52 (7.28 – 10.92) (n=175)	9.80 (7.84 – 11.76) (n=182)	0.07
Infant venous blood (Two months)	6.72 (5.04 – 8.12) (n=146)	5.88 (4.76 – 7.28) (n=182)	0.003
Child venous blood (14-16 months)	13.17 (10.64 – 15.69) (n=68)	12.9 (10.64 – 15.13) (n=73)	0.97
Maternal venous blood (Birth)	7.84 (6.16 – 9.52) (n=215)	8.40 (6.72 – 10.08) (n=225)	0.03
**Copenhagen Baby Heart - Urea concentration (mg/dL)**			
Umbilical cord blood	9.24 (7.56 – 11.20) (n=6,207)	9.24 (7.84 – 11.20) (n=6,482)	<0.001

Urea concentrations are stratified by sex and presented as median (interquartile range) and n: number of individuals. P-values by Mann-Whitney U test. To convert mg/dL to mmol/L multiply by 0.357.

© 2022 Mohr Lytsen RM et al. *JAMA Network Open*.

eTable 7. Reference Intervals for Creatinine Concentrations in Children's Venous Blood

	Newborn (GA 37-39)	Newborn (GA 40-42)	Infant (2 months)	Child (14-16 months)
**Creatinine concentration (mg/dL)**				
Model 1 (n)	0.49 – 1.13(171)	0.52 – 1.32 (182)	0.10 – 0.32 (313)	0.18 – 0.40 (139)
Model 2 (n)	0.51 – 1.10 (167)	0.55 – 1.19 (176)	0.12 – 0.31 (305)	0.18 – 0.34 (131)
Model 3 (n)	0.49 – 1.08 (145)	0.54 – 1.32 (167)	0.11 – 0.33 (276)	0.17 – 0.40 (122)
Model 4 (n)	0.54 – 1.08 (144)	0.57 – 1.19 (162)	0.13 – 0.31 (268)	0.18 – 0.34 (115)
Existing local reference intervals (Rigshospitalet, Copenhagen, Denmark)	0.42 – 0.92 (5)<br>(0 – 1 days)		0.19 – 0.46 (95)<br>(14 days – 2 months)	0.17 – 0.35 (45)<br>(1 – 3 years)

Reference intervals are presented as 2.5 and 97.5 percentiles (number of individuals) and are from newborn venous blood at the time of birth (2016-2018), infant venous blood at 2 months and child venous blood at 14-16 months after birth (2016-2020). Newborn reference intervals from the time of birth are stratified by gestational age. In model 1 the 2.5 and 97.5 percentiles unadjusted are shown. Model 2 is the 2.5 and 97.5 percentiles adjusted for outliers as recommended by Tietz. Model 3 is the 2.5 and 97.5 percentiles adjusted for the following exclusion criteria: preeclampsia, maternal diabetes, maternal kidney disease, congenital kidney deformity, maternal BMI >35 and neonates born small for gestational age. In model 4 the 2.5 and 97.5 percentiles adjusted for outliers and exclusion criteria are shown. The existing local reference intervals are from the Clinical Biochemical Department on Rigshospitalet, Copenhagen, Denmark, and are determined from Boer et al. (0 – 1 days and 14 days – 2 months)[18] and Ceriotti et al. (1 – 3 years)[19]. To convert mg/dL to µmol/L multiply by 88.4. GA: Gestational age.

© 2022 Mohr Lytsen RM et al. *JAMA Network Open*.

eTable 8. Reference Intervals for Urea Concentrations in Children's Venous Blood

	Newborn (GA 37-39)	Newborn (GA 40-42)	Infant (2 months)	Child (14-16 months)
**Urea concentration (mg/dL)**				
Model 1 (n)	5.32 – 16.06 (172)	5.60 – 15.88 (185)	3.14 – 10.64 (328)	6.60 – 19.17 (141)
Model 2 (n)	5.32 – 14.79 (167)	5.60 – 14.85 (179)	3.14 – 10.64 (328)	8.12 – 17.84 (131)
Model 3 (n)	5.32 – 15.13 (146)	5.60 – 16.08 (171)	3.08 – 10.08 (288)	6.48 – 19.26 (124)
Model 4 (n)	5.32 – 14.67 (144)	5.60 – 14.85 (165)	3.08 – 10.08 (288)	8.12 – 17.95 (116)
Existing local reference intervals (Rigshospitalet, Copenhagen, Denmark)	3.92 – 15.1 (0 – 2 months)		5.04 – 15.1 (2 months – 2 years)	

Reference intervals are presented as 2.5 and 97.5 percentiles (number of individuals) and are from newborn venous blood at the time of birth (2016-2018), infant venous blood at 2 months and child venous blood at 14-16 months after birth (2016-2020). Newborn reference intervals from the time of birth are stratified by gestational age. In model 1 the 2.5 and 97.5 percentiles unadjusted are shown. Model 2 is the 2.5 and 97.5 percentiles adjusted for outliers as recommended by Tietz. Model 3 is the 2.5 and 97.5 percentiles adjusted for the following exclusion criteria; preeclampsia, maternal diabetes, maternal kidney disease, congenital kidney deformity, maternal BMI >35 and neonates born small for gestational age. In model 4 the 2.5 and 97.5 percentiles adjusted for outliers and exclusion criteria are shown. The existing local reference intervals are from the Clinical Biochemical Department on Rigshospitalet, Copenhagen, Denmark. To convert mg/dL to µmol/L multiply by 0.357. GA: Gestational age.

© 2022 Mohr Lytsen RM et al. *JAMA Network Open*.

eTable 9. New and Existing Reference Intervals for Creatinine and Urea Concentrations and Number of Children From the COMPARE Study With Concentrations Higher and Lower Than the Reference Limits

	Newborn (GA 37-39)	Newborn (GA 40-42)	Infant (2 months)	Child (14-16 months)
**Creatinine, mg/dL[a] (n)**	0.54 – 1.08 (144)	0.57 – 1.19 (162)	0.13 – 0.31 (268)	0.18 – 0.34 (115)
Children outside reference limits, No. (%)	5 (2.9%) and 9 (5.3%)	5 (2.8%) and 7 (3.9%)	13 (4.2%) and 10 (3.2%)	3 (2.2%) and 9 (6.5%)
**Existing local reference intervals (Rigshospitalet, Copenhagen, Denmark)**	0.42 – 0.92 (5)<br>(0 – 1 days)		0.19 – 0.46 (95)<br>(14 days – 2 months)	0.17 – 0.35 (45)<br>(1 – 3 years)
Children outside reference limits, No. (%)	2 (0.6%) and 93 (26.4%)		86 (27.5%) and 0	1 (0.7%) and 8 (5.8%)
**Urea, mmol/L[b] (n)**	5.32 – 14.67 (144)	5.60 – 14.85 (165)	3.08 – 10.08 (288)	8.12 – 17.95 (116)
Children outside reference limits, No. (%)	3 (1.7%) and 9 (5.2%)	3 (1.6%) and 9 (4.9%)	4 (1.2%) and 10 (3.1%)	6 (4.3%) and 8 (5.7%)
**Existing local reference intervals (Rigshospitalet, Copenhagen, Denmark)**	3.92 – 15.1<br>(0 – 2 months)		5.04 – 15.1<br>(2 months – 2 years)	
Children outside reference limits, No. (%)	2 (0.6%) and 11 (3.1%)		82 (25.0%) and 0	2 (1.4%) and 32 (22.7%)

Reference intervals are presented as 2.5 and 97.5 percentiles (number of individuals) adjusted for outliers and exclusion criteria and are from newborn venous blood at the time of birth (2016-2018), infant venous blood at 2 months and child venous blood at 14-16 months after birth (2016-2020). Newborn reference intervals from the time of birth are stratified by gestational age. Outliers were adjusted as recommended by Tietz. Exclusion criteria were preeclampsia, maternal diabetes, maternal kidney disease, congenital kidney deformity, maternal BMI >35 and neonates born small for gestational age. The existing local reference intervals are from the Clinical Biochemical Department on Rigshospitalet, Copenhagen, Denmark, and are determined from Boer et al. (0 – 1 days and 14 days – 2 months)[18] and Ceriotti et al. (1 – 3 years)[19] for creatinine. The number of children concentrations outside the reference intervals are given for each interval. GA: Gestational age. [a] To convert mg/dL to µmol/L multiply by 88.4. [b] To convert mg/dL to µg/dL multiply by 0.357.

© 2022 Mohr Lytsen RM et al. *JAMA Network Open*.

eTable 10. Reference Intervals for Creatinine Concentrations in Umbilical Cord Blood

	GA 37-39	GA 40-42	Combined
**Creatinine concentration (µmol/L)**			
Model 1 (n)	0.38 – 1.06 (4,814)	0.42 – 1.14 (6,980)	0.41 – 1.11 (11,794)
Model 2 (n)	0.43 – 0.96 (4,606)	0.49 – 1.01 (6,630)	0.45 – 0.98 (11,230)
Model 3 (n)	0.37 – 1.04 (3,959)	0.43 – 1.12 (6,362)	0.40 – 1.09 (10,321)
Model 4 (n)	0.43 – 0.95 (3,805)	0.49 – 1.01 (6,062)	0.45 – 0.98 (9,861)
Existing local reference intervals (Rigshospitalet, Copenhagen, Denmark)	No existing reference intervals		

Reference intervals are presented as 2.5 and 97.5 percentiles (number of individuals) and are from umbilical cord blood (2016-2018) and are presented stratified by gestational age 37-39 weeks and 40-42 weeks and combined. In model 1 the 2.5 and 97.5 percentiles unadjusted are shown. Model 2 is the 2.5 and 97.5 percentiles adjusted for outliers as recommended by Tietz. Model 3 is the 2.5 and 97.5 percentiles adjusted for the following exclusion criteria: preeclampsia, maternal diabetes, maternal kidney disease, congenital kidney deformity, maternal BMI >35 and neonates born small for gestational age. In model 4 the 2.5 and 97.5 percentiles adjusted for outliers and exclusion criteria are shown. To convert mg/dL to µmol/L multiply by 88.4. GA: Gestational age.

© 2022 Mohr Lytsen RM et al. *JAMA Network Open*.

eTable 11. Reference Intervals for Urea Concentrations in Umbilical Cord Blood

	GA 37-39	GA 40-42	Combined
**Urea concentration (mmol/L)**			
Model 1 (n)	5.04 – 15.97 (5,017)	5.32 – 16.25 (7,282)	5.04 – 16.25 (12,299)
Model 2 (n)	5.04 – 14.29 (4,836)	5.32 – 14.29 (7,012)	5.32 – 14.29 (11,848)
Model 3 (n)	4.76 – 15.41 (4,143)	5.32 – 15.97 (6,635)	5.04 – 15.97 (10,778)
Model 4 (n)	5.04 – 14.01 (4,032)	5.32 – 14.29 (6,407)	5.32 – 14.29 (10,439)
Existing local reference intervals (Rigshospitalet, Copenhagen, Denmark)	No existing reference intervals		

Reference intervals are presented as 2.5 and 97.5 percentiles (number of individuals) and are from umbilical cord blood (2016-2018) and are presented stratified by gestational age 37-39 weeks and 40-42 weeks and combined. In model 1 the 2.5 and 97.5 percentiles unadjusted are shown. Model 2 is the 2.5 and 97.5 percentiles adjusted for outliers as recommended by Tietz. Model 3 is the 2.5 and 97.5 percentiles adjusted for the following exclusion criteria: preeclampsia, maternal diabetes, maternal kidney disease, congenital kidney deformity, maternal BMI >35 and neonates born small for gestational age. In model 4 the 2.5 and 97.5 percentiles adjusted for outliers and exclusion criteria are shown. To convert mg/dL to mmol/L multiply by 0.357. GA: Gestational age.

© 2022 Mohr Lytsen RM et al. *JAMA Network Open*.

eFigure 1. Study Flowchart

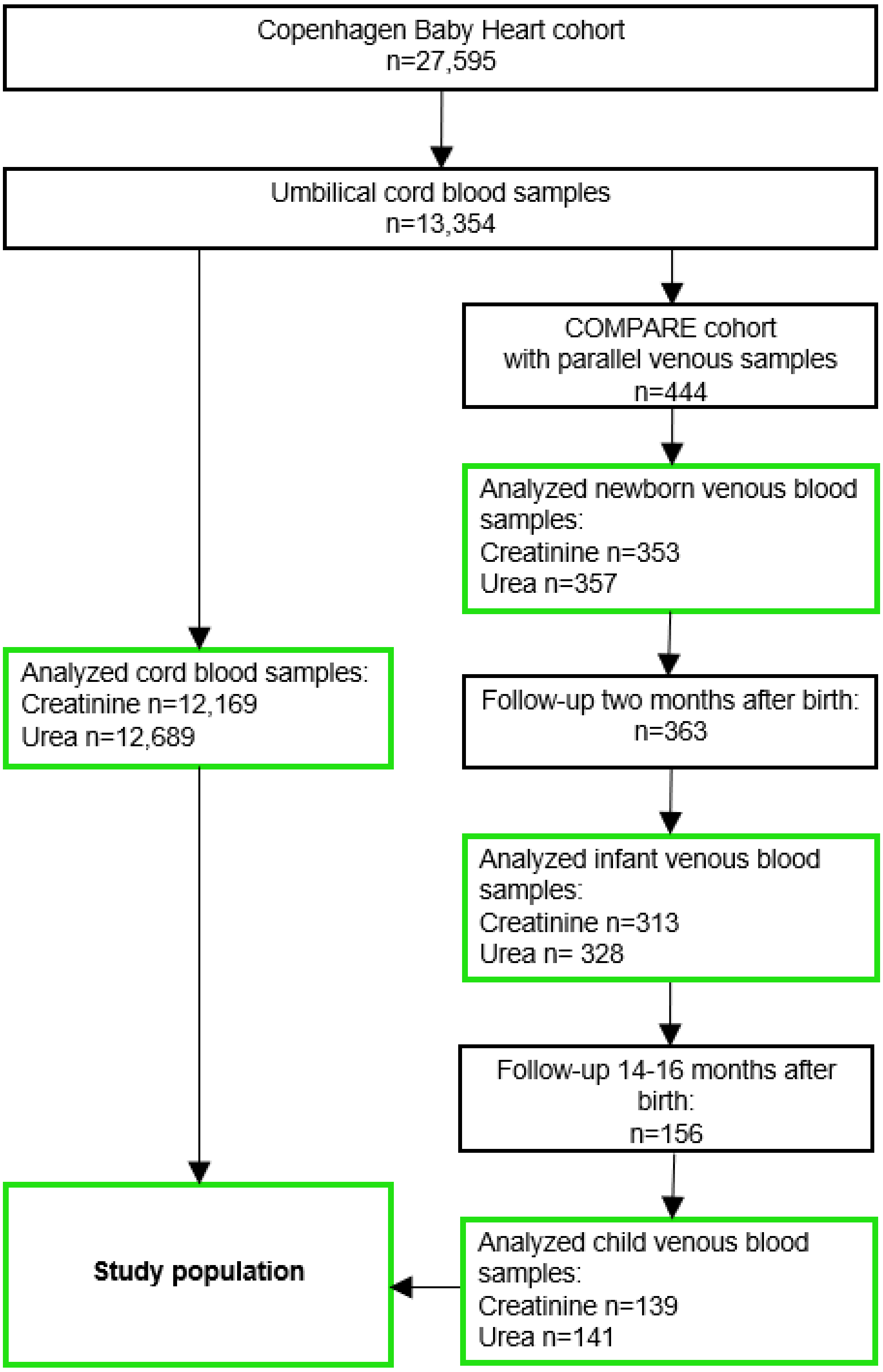

Flowchart illustrating the recruitment and exclusion of participants in the Copenhagen Baby Heart Study and the COMPARE study cohorts. Numbers (n) of individuals and blood samples analyzed for creatinine and urea in umbilical cord blood and child venous blood at birth, two months and 14-16 months after birth are shown.

© 2022 Mohr Lytsen RM et al. *JAMA Network Open*.

eFigure 2. Consequences of Maternal Comorbidities for the Risk of High Urea Concentrations in the Newborn

Umbilical cord blood urea concentration > reference interval (14.29 mg/dL)

	Unadjusted				Multifactorially adjusted			
**Exposure**	**Individuals**	**Events**	**Odds ratio (CI)**	**P-value**	**Individuals**	**Events**	**Odds ratio (CI)**	**P-value**
Preeclampsia								
No	11,992	589	1.00 (reference)		11,779	580	1.00 (reference)	
Yes	691	115	3.87 (3.11, 4.80)	<0.001	681	112	3.91 (3.12, 4.91)	<0.001
Maternal diabetes								
No	12,310	658	1.00 (reference)		12,091	647	1.00 (reference)	
Yes	373	46	2.49 (1.81, 3.43)	<0.001	369	45	2.76 (1.97, 3.86)	<0.001
Maternal hypertension								
No	12,337	665	1.00 (reference)		12,116	653	1.00 (reference)	
Yes	346	39	2.23 (1.58, 3.14)	<0.001	344	39	2.21 (1.57, 3.13)	<0.001
Maternal Body Mass Index >=25								
No	9,398	506	1.00 (reference)		9,225	497	1.00 (reference)	
Yes	3,270	199	1.14 (0.96, 1.35)	0.13	3,214	195	1.19 (1.00, 1.41)	0.05
Maternal smoking								
No	12,008	675	1.00 (reference)		11,794	662	1.00 (reference)	
Yes	392	16	0.71 (0.43, 1.19)	0.19	382	16	0.72 (0.43, 1.19)	0.20

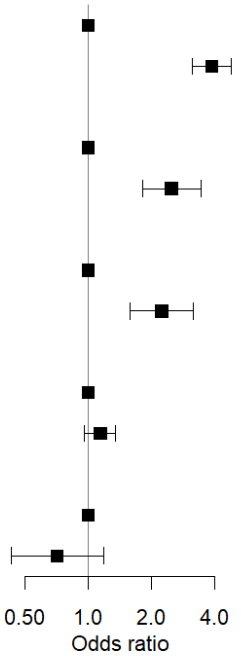

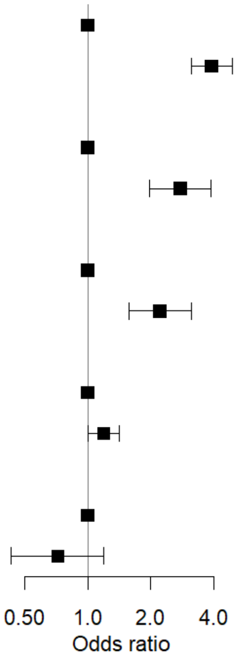

Forest plot showing odds ratios of the newborn having an umbilical cord blood urea concentration above the upper reference limit as a function of maternal comorbidities. Odds ratios, confidence intervals and p-values are from logistic regression. Individuals represent the number of newborns with or without the maternal comorbidity registered. Events represent the number of newborns with a urea concentration above the upper reference limit. In the multifactorially adjusted model odds ratios were adjusted for maternal age at birth, child's sex, gestational age, birth weight, birth length, multiple births, and placental-fetal weight ratio above or below the median. To convert mg/dL to mmol/L multiply by 0.357.

© 2022 Mohr Lytsen RM et al. *JAMA Network Open*.

eFigure 3. Consequences of Maternal Preeclampsia for the Risk of High Creatinine Concentrations in Newborn Venous Blood at Birth

	Unadjusted				Multifactorially adjusted			
**Creatinine concentration at birth**	**Individuals**	**Events**	**Odds ratio (CI)**	**P-value**	**Individuals**	**Events**	**Odds ratio (CI)**	**P-value**
> upper reference (1.14 mg/dL)								
No preeclampsia	330	10	1.00 (reference)		325	10	1.00 (reference)	
Preeclampsia	23	4	6.74 (1.93, 23.5)	<0.01	22	3	9.40 (1.68, 52.5)	0.01
> 90th percentile (1.06 mg/dL)								
No preeclampsia	330	26	1.00 (reference)		325	25	1.00 (reference)	
Preeclampsia	23	6	4.13 (1.50, 11.4)	<0.01	22	5	4.29 (1.32, 13.9)	0.02
> 80th percentile (0.96 mg/dL)								
No preeclampsia	330	61	1.00 (reference)		325	60	1.00 (reference)	
Preeclampsia	23	9	2.83 (1.17, 6.85)	0.02	22	8	3.10 (1.14, 8.46)	0.03
> 50th percentile (0.80 mg/dL)								
No preeclampsia	330	161	1.00 (reference)		325	159	1.00 (reference)	
Preeclampsia	23	13	1.36 (0.58, 3.20)	0.48	22	12	1.64 (0.63, 4.27)	0.32

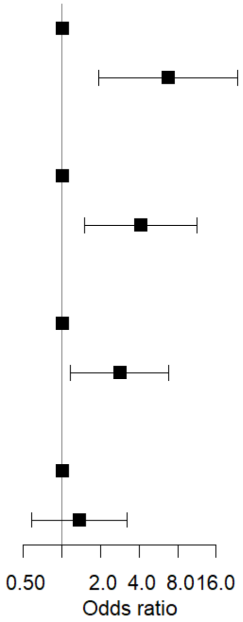

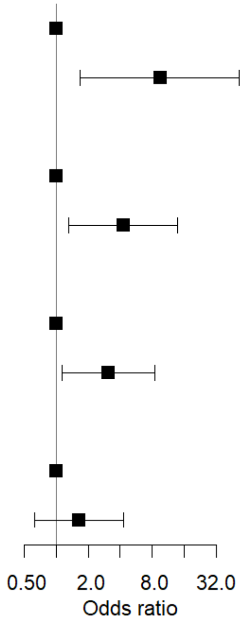

Forest plot showing odds ratios of the newborn having a venous blood creatinine concentration at birth above the upper reference limit (1.14 mg/dL), above the 90th percentile (1.06 mg/dL), above the 80th percentile (0.96 mg/dL) and above the 50th percentile (0.80 mg/dL) as a function of maternal preeclampsia. Odds ratios, confidence intervals and p-values are from logistic regression. Individuals represent the number of newborns with or without a mother with registered preeclampsia. Events represent the number of newborns with a creatinine concentration above the cut-off value. In the multifactorially adjusted model odds ratios were adjusted for maternal age at birth, child's sex, gestational age, birth weight, birth length, multiple births, and placental-fetal weight ratio above or below the median. To convert mg/dL to µmol/L multiply by 88.4.

© 2022 Mohr Lytsen RM et al. *JAMA Network Open*.

eFigure 4. Consequences of Maternal Preeclampsia for the Risk of High Creatinine Concentrations in Infant Venous Blood at 2 Months

	Unadjusted				Multifactorially adjusted			
**Creatinine concentration at two months**	**Individuals**	**Events**	**Odds ratio (CI)**	**P-value**	**Individuals**	**Events**	**Odds ratio (CI)**	**P-value**
> upper reference (0.31 mg/dL)								
No preeclampsia	293	10			290	10		
Preeclampsia	20	0	No events		19	0	No events	
> 90th percentile (0.27 mg/dL)								
No preeclampsia	293	27	1.00 (reference)		290	27	1.00 (reference)	
Preeclampsia	20	3	1.74 (0.48, 6.31)	0.40	19	3	1.75 (0.46, 6.63)	0.41
> 80th percentile (0.25 mg/dL)								
No preeclampsia	293	56	1.00 (reference)		290	56	1.00 (reference)	
Preeclampsia	20	6	1.81 (0.67, 4.93)	0.24	19	6	1.76 (0.62, 4.98)	0.28
> 50th percentile (0.21 mg/dL)								
No preeclampsia	293	126	1.00 (reference)		290	126	1.00 (reference)	
Preeclampsia	20	9	1.08 (0.44, 2.70)	0.86	19	9	1.17 (0.45, 3.05)	0.74

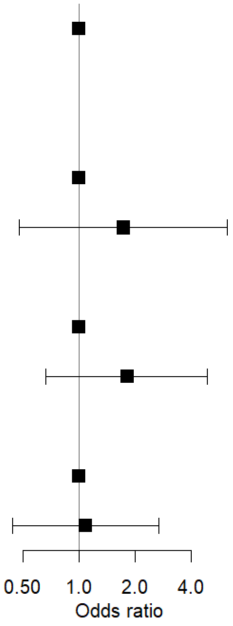

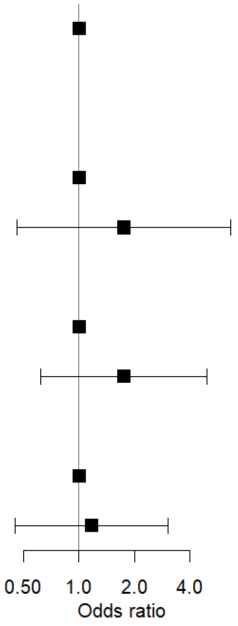

Forest plot showing the odds ratio of the infant having a venous blood creatinine concentration at two months above the upper reference limit (0.31 mg/dL), above the 90th percentile (0.27 mg/dL), above the 80th percentile (0.25 mg/dL) and above the 50th percentile (0.21 mg/dL) as a function of maternal preeclampsia. Odds ratios, confidence intervals and p-values are from logistic regression. Individuals represent the number of infants with or without a mother with registered preeclampsia. Events represent the number of infants with a creatinine concentration above the cut-off value. In the multifactorially adjusted model odds ratios were adjusted for maternal age at birth, child's sex, gestational age, birth weight, birth length, multiple births, and placental-fetal weight ratio above or below the median. To convert mg/dL to µmol/L multiply by 88.4.

© 2022 Mohr Lytsen RM et al. *JAMA Network Open*.

eFigure 5. Consequences of Maternal Preeclampsia for Urea Concentrations in Newborn Venous Blood at Birth

	Unadjusted				Multifactorially adjusted			
**Urea concentration at birth**	**Individuals**	**Events**	**Odds ratio (CI)**	**P-value**	**Individuals**	**Events**	**Odds ratio (CI)**	**P-value**
> upper reference (14.9 mg/dL)								
No preeclampsia	334	15	1.00 (reference)		330	15	1.00 (reference)	
Preeclampsia	23	2	2.03 (0.43, 9.45)	0.37	22	1	1.14 (0.13, 9.71)	0.91
> 90th percentile (13.7 mg/dL)								
No preeclampsia	334	29	1.00 (reference)		330	28	1.00 (reference)	
Preeclampsia	23	4	2.21 (0.71, 6.95)	0.17	22	3	1.94 (0.51, 7.29)	0.32
> 80th percentile (11.8 mg/dL)								
No preeclampsia	334	62	1.00 (reference)		330	61	1.00 (reference)	
Preeclampsia	23	8	2.34 (0.95, 5.76)	0.07	22	7	2.46 (0.91, 6.67)	0.08
> 50th percentile (9.52 mg/dL)								
No preeclampsia	334	160	1.00 (reference)		330	159	1.00 (reference)	
Preeclampsia	23	16	2.49 (1.00, 6.20)	0.05	22	15	3.44 (1.25, 9.47)	0.02

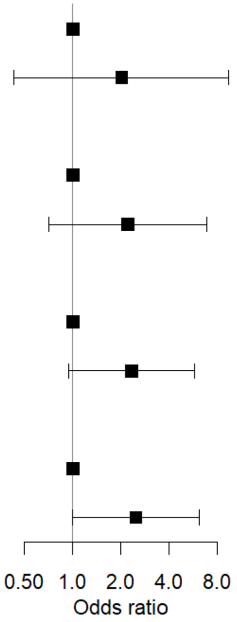

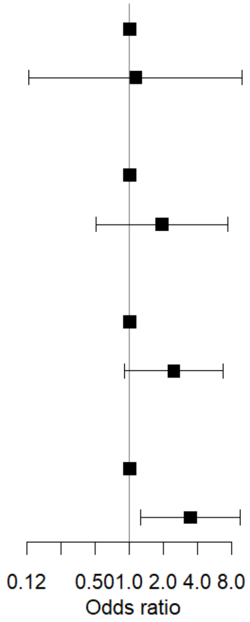

Forest plot showing the impact of maternal preeclampsia on the odds ratio of the newborn having a venous blood urea concentration at birth above the upper reference limit (14.9 mg/dL), above the 90th percentile (13.7 mg/dL), above the 80th percentile (11.8 mg/dL) and above the 50th percentile (9.52 mg/dL) respectively. Odds ratios, confidence intervals and p-values are from logistic regression. Individuals represent the number of newborns with or without registration of preeclampsia. Events represent the number of newborns with a urea concentration above the cut-off value. In the multifactorially adjusted model odds ratios were adjusted for maternal age at birth, child's sex, gestational age, birth weight, birth length, multiple births, and placental-fetal weight ratio above or below the median. To convert mg/dL to mmol/L multiply by 0.357.

© 2022 Mohr Lytsen RM et al. *JAMA Network Open*.

eFigure 6. Consequences of Maternal Preeclampsia for Urea Concentrations in Infant Venous Blood at 2 Months

	Unadjusted				Multifactorially adjusted			
**Urea concentration at two months**	**Individuals**	**Events**	**Odds ratio (CI)**	**P-value**	**Individuals**	**Events**	**Odds ratio (CI)**	**P-value**
> upper reference (10.1 mg/dL)								
No preeclampsia	334	15	1.00 (reference)		330	15	1.00 (reference)	
Preeclampsia	23	2	1.66 (0.20, 13.7)	0.64	22	1	1.98 (0.20, 19.3)	0.56
> 90th percentile (8.68 mg/dL)								
No preeclampsia	334	29	1.00 (reference)		330	28	1.00 (reference)	
Preeclampsia	23	4	1.14 (0.25, 5.16)	0.87	22	3	1.33 (0.26, 6.67)	0.73
> 80th percentile (8.12 mg/dL)								
No preeclampsia	334	62	1.00 (reference)		330	61	1.00 (reference)	
Preeclampsia	23	8	0.82 (0.23, 2.88)	0.75	22	7	0.91 (0.24, 3.43)	0.89
> 50th percentile (6.16 mg/dL)								
No preeclampsia	334	160	1.00 (reference)		330	159	1.00 (reference)	
Preeclampsia	23	16	1.28 (0.53, 3.10)	0.59	22	15	1.44 (0.56, 3.66)	0.45

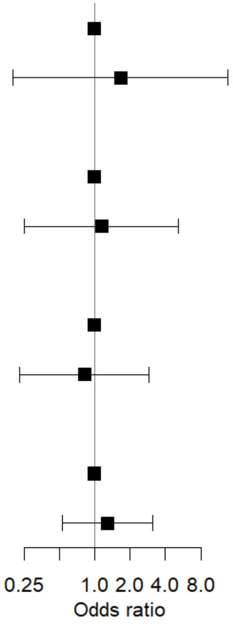

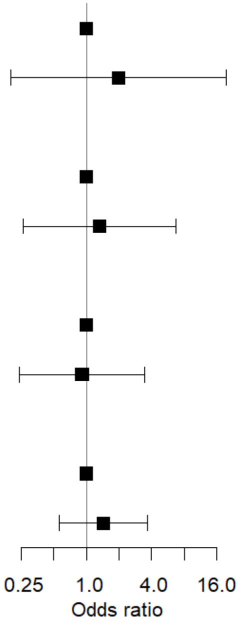

Forest plot showing the impact of maternal preeclampsia on the odds ratio of the infant having a venous blood urea concentration at two months above the upper reference limit (10.1 mg/dL), above the 90th percentile (8.68 mg/dL), above the 80th percentile (8.12 mg/dL) and above the 50th percentile (6.16 mg/dL). Odds ratios, confidence intervals and p-values are from logistic regression. Individuals represent the number of infants with or without registration of preeclampsia. Events represent the number of infants with a urea concentration above the cut-off value. In the multifactorially adjusted model odds ratios were adjusted for maternal age at birth, child's sex, gestational age, birth weight, birth length, multiple births, and placental-fetal weight ratio above or below the median. To convert mg/dL to mmol/L multiply by 0.357.

© 2022 Mohr Lytsen RM et al. *JAMA Network Open*.

eReferences

1. Sillesen AS, Raja AA, Pihl C, et al. Copenhagen Baby Heart Study: a population study of newborns with prenatal inclusion. *Eur J Epidemiol*. 2019;34(1):79-90. doi:10.1007/s10654-018-0448-y.
2. Rigshospitalets Labportal [Online]. Available from: https://labportal.rh.dk/.
3. Marsál K, Persson PH, Larsen T, Lilja H, Selbing A, Sultan B. Intrauterine growth curves based on ultrasonically estimated foetal weights *Acta Paediatr*. 1996;85(7):843-848. doi:10.1111/j.1651-2227.1996.tb14164.x.
4. Filler G, Lopes L, Harrold J, Bariciak E. β-trace protein may be a more suitable marker of neonatal renal function. *Clin Nephrol*. 2014;81(4):269-276. doi:10.5414/CN108089
5. Davis BM, Miller RK, Brent RL, Koszalka TR. Materno-Fetal Transport of Creatine in the Rat. *Biol Neonate*. 1978;33(1-2):43-54. doi:10.1159/000241050
6. Lao TT, Loong EP, Chin RK, Lam YM. Renal function in the newborn. Newborn creatinine related to birth weight, maturity and maternal creatinine. *Gynecol Obstet Invest*. 1989;28(2):70-72. doi:10.1159/000293517
7. Nava S, Bocconi L, Zuliani G, Kustermann A, Nicolini U. Aspects of fetal physiology from 18 to 37 weeks' gestation as assessed by blood sampling. *Obstet Gynecol*. 1996;87(6):975-980. doi:10.1016/0029-7844(96)00056-7
8. Filler G, Bhayana V, Schott C, Díaz-González de Ferris ME. How should we assess renal function in neonates and infants? *Acta Paediatrica*. 2021;110(3):773-780. doi:10.1111/apa.15557
9. Falcão MC, Okay Y, Ramos JL. Relationship between plasma creatinine concentration and glomerular filtration in preterm newborn infants. *Rev Hosp Clin Fac Med Sao Paulo*. 1999;54(4):121-126. doi:10.1590/s0041-87811999000400004
10. Miall LS, Henderson MJ, Turner AJ, et al. Plasma Creatinine Rises Dramatically in the First 48 Hours of Life in Preterm Infants. *Pediatrics*. 1999;104(6)e76. doi:10.1542/peds.104.6.e76.
11. Schwartz GJ, Feld LG, Langford DJ. A simple estimate of glomerular filtration rate in full-term infants during the first year of life. *J Pediatr*. 1984;104(6):849-854. doi:10.1016/s0022-3476(84)80479-5.
12. Pottel H, Vrydags N, Mahieu B, Van dewynckele E, Croes K, Martens F. Establishing age/sex related serum creatinine reference intervals from hospital laboratory data based on different statistical methods. *Clin Chim Acta*. 2008;396(1-2):49-55. doi:10.1016/j.cca.2008.06.017
13. Guignard JP, Drukker A. Why Do Newborn Infants Have a High Plasma Creatinine? *Pediatrics*. 1999;103(4):e49. doi:10.1542/peds.103.4.e49
14. Sang-Yoon L, Jung-Eun M, Park S. Longitudinal changes in serum creatinine levels and urinary biomarkers in late preterm infants during the first postnatal week: Association with acute kidney

injury and treatment with aminoglycoside. *Children (Basel)*. 2021;8(10):896. doi:10.3390/children8100896

15. Bueva A, Guignard JP. Renal Function in Preterm Neonates. *Pediatr Res*. 1994;36(5):572-577. doi:10.1203/00006450-199411000-00005
16. Treiber M, Pečovnik Balon B, Gorenjak M. A new serum cystatin C formula for estimating glomerular filtration rate in newborns. *Pediatr Nephrol*. 2015;30(8):1297–1305. doi:10.1007/s00467-014-3029-7
17. Kher K, Mistry K. Assessment of Glomerular and Tubular Function. *Curr Pediatr Rev*. 2014;10(2):142-150. doi:10.2174/157339631002140513102352
18. Boer DP, de Rijke YB, Hop WC, et al. Reference values for serum creatinine in children younger than 1 year of age. *Pediatr Nephrol*. 2010;25(10):2107–2113. doi:10.1007/s00467-010-1533-y
19. Ceriotti F, Boyd JC, Klein G, et al. Reference intervals for serum creatinine concentrations: assessment of available data for global application. *Clin Chem*. 2008;54(3):559-566. doi:10.1373/clinchem.2007.099648

© 2022 Mohr Lytsen RM et al. *JAMA Network Open*.
